# Efficacy of *Bifidobacterium lactis* BLa80 in preventing early childhood eczema and respiratory infections via gut microbiome and immune modulation

**DOI:** 10.3389/fnut.2026.1727191

**Published:** 2026-05-28

**Authors:** Ke Chen, Shanshan Jin, Yang Nie, Nianyang He, Haixia Chen, Jie Yuan, Xiaohui Li, Min-Tze Liong

**Affiliations:** 1 Department of Clinical Nutrition, Chengdu Women's & Children's Central Hospital, School of Medicine, University of Electronic Science and Technology of China, Chengdu, Sichuan, China; 2 Department of Child Health Care, Chongzhou Maternal and Child Health Care Hospital, Chongzhou, Sichuan, China; 3 Department of Child Health Care, Xindu Maternal and Child Health Care Hospital, Chengdu, Sichuan, China; 4 Baoxing Central for Disease Prevention and Control, Yaan, Sichuan, China; 5 Laboratory of Microbiology, Immunology, and Metabolism, DiPROBIO (Shanghai) Co., Ltd., Shanghai, China

**Keywords:** *Bifidobacterium animalis* subsp. *lactis* BLa80, child, gut microbiota, probiotic, randomized controlled trial

## Abstract

**Background:**

Early childhood is a critical period for immune development, with eczema and respiratory infections representing common health challenges. This study investigated the efficacy of *Bifidobacterium animalis* subsp. *lactis* BLa80 in reducing these conditions potentially through gut microbiome modulation.

**Methods:**

In a randomized, double-blind, placebo-controlled trial, 360 formula-fed infants and children aged below 3 years old with elevated allergy risk received daily *B. lactis* BLa80 (5 × 10^9^ CFU) or placebo for 180 days. Primary outcomes included eczema incidence and symptom burden, with secondary outcomes assessing respiratory infections, gastrointestinal symptoms, gut microbiota composition (16S rRNA sequencing), functional pathways (KEGG analysis), and fecal immune markers (ELISA).

**Results:**

The probiotic group demonstrated significantly reduced eczema incidence (27.6% vs. 69.5%, RR: 0.398, *p* < 0.001) and upper respiratory tract infections (19.4% vs. 42.5%, RR: 0.457, *p* < 0.001). Significant reductions were observed in symptom burden, including nasal congestion, vomiting, milk aspiration, and irritability. Microbiota profiling showed enrichment of beneficial taxa (*Akkermansia, Fusicatenibacter*) with enhanced metabolic pathways including tryptophan metabolism, vitamin biosynthesis, and xenobiotic degradation. Immunological profiling showed maintained human beta-defensin-2 (*p* = 0.005), increased secretory IgA (*p* < 0.001), and reduced calprotectin (*p* < 0.001).

**Conclusions:**

*B. lactis* BLa80 supplementation effectively reduces eczema and respiratory infections associated with gut microbiome remodeling that may enhance barrier function, immune regulation, and metabolic capacity, supporting its use as a preventive nutritional strategy in early childhood.

**Clinical Trial Registration:**

ChiCTR2300074956.

## 1 Introduction

The early childhood period represents a critical window for immune development and microbial colonization, with the establishment of the gut microbiome playing a pivotal role in shaping long-term health outcomes (1, 2). During this formative stage, the immature immune system is particularly vulnerable to dysregulation, manifesting in a rising prevalence of allergic diseases and recurrent infections (3). Among these, eczema and respiratory tract infections constitute significant pediatric health burdens, affecting quality of life, healthcare utilization, and potentially leading to the development of other allergic conditions in later life through the “atopic march” (4, 5).

The gut microbiome has emerged as a central regulator of immune homeostasis, with its composition and metabolic activity influencing systemic immune responses through multiple pathways. The concept of the gut-skin axis and gut-lung axis has gained substantial scientific support, suggesting that gut microbial communities can modulate inflammatory processes in distant organs (6). *Bifidobacterium* species, as early colonizers of the infant gut, have attracted particular interest due to their immunomodulatory properties and association with reduced allergy risk (7). However, not all probiotic strains demonstrate equal efficacy, and strain-specific effects are increasingly recognized as crucial determinants of clinical outcomes (8).

Current evidence regarding probiotic interventions for allergy prevention presents considerable heterogeneity in outcomes (9). While some studies demonstrate promising results, others show limited benefits, highlighting the need for well-designed trials investigating specific probiotic strains with comprehensive mechanistic insights (10). Furthermore, many existing studies focus primarily on clinical endpoints without integrating microbiome profiling, predicted functional analysis, and immunological assessment to elucidate the underlying microbial and immunological mechanisms (11). This gap is particularly relevant for understanding how specific probiotic strains remodel the gut ecosystem to exert their health benefits (12).

*Bifidobacterium animalis* subsp. *lactis* BLa80 is a novel probiotic strain isolated for its potential immunomodulatory properties. Previous *in vitro* studies have demonstrated its ability to enhance epithelial barrier function and modulate immune responses, suggesting its potential utility in allergy prevention (13–15). However, robust clinical evidence from randomized controlled trials is necessary to establish its efficacy and understand its mechanism of action in pediatric populations.

This randomized, double-blind, placebo-controlled trial was therefore designed to address these critical gaps in knowledge. We aimed to investigate the efficacy of BLa80 supplementation in reducing the incidence of eczema and respiratory infections in high-risk infants and young children, while simultaneously employing 16S rRNA gene sequencing, predicted functional profiling, and immunological analyses to elucidate the mechanistic basis for any observed clinical effects. By integrating detailed clinical phenotyping with microbiome and immune profiling, this study seeks to provide not only evidence of clinical efficacy but also a comprehensive understanding of how BLa80 remodels the gut ecosystem to promote immune health in early childhood.

The findings from this study will contribute valuable insights into the strain-specific effects of probiotics and provide a mechanistic framework for understanding how targeted microbial interventions can modulate immune development during this critical period. This knowledge is essential for developing evidence-based nutritional strategies for primary prevention of allergic diseases and respiratory infections in pediatric populations.

## 2 Methods

### 2.1 Study design and ethical approval

This randomized, double-blind, placebo-controlled clinical trial was conducted in accordance with the Declaration of Helsinki, ICH-GCP standards, and CONSORT 2010 reporting guidelines. Participants were randomly assigned (1:1) to receive either the probiotic *Bifidobacterium animalis* subsp. *lactis* BLa80 or placebo for 180 days. Randomization was performed by an independent statistician using a computer-generated sequence (block size = 4). Allocation concealment was maintained through identical packaging and coded labeling of intervention materials. Both participants and investigators were blinded to group allocation until data analysis was complete.

The study protocol was approved by the Ethics Committee of Chengdu Women and Children's Central Hospital [Scientific Research Ethics Review Approval No. 2023(72)] and registered at the Chinese Clinical Trial Registry (ChiCTR2300074956). Written informed consent was obtained from parents or legal guardians prior to enrollment.

### 2.2 Participants and eligibility criteria

Full-term, exclusively formula-fed infants and young children aged 15 days to 36 months were eligible for enrollment. Participants were required to have been born between 37 and 42 weeks of gestation with a birth weight ranging from 2,500 g to less than 4,000 g. Additional inclusion criteria comprised an allergy risk score of ≥6 points as assessed by the standardized “Infant and Child Allergy Risk Assessment Scale” developed by the Chinese Center for Disease Control and Prevention Center for Maternal and Child Health. Furthermore, eligible participants had to be under regular follow-up at a local Maternal and Child Health Hospital's Child Health Unit and receiving standard feeding guidance from child healthcare practitioners. Mothers of participants were required to have no history of diagnosed metabolic diseases (including diabetes) or chronic infectious diseases (including hepatitis B or HIV). Crucially, participants were excluded if they presented with any clinician-diagnosed allergic diseases at enrollment, including but not limited to eczema, asthma, allergic proctocolitis, allergic rhinitis, or hay fever. Children must not use antibiotics within the past month or probiotic supplements within the previous 3 months.

Participants were excluded from the study based on the following criteria: (1) history of birth asphyxia or neonatal intensive care unit hospitalization; (2) presence of congenital anomalies or birth defects; (3) maternal history of high-risk obstetric conditions including pregnancy-induced hypertension, eclampsia, preeclampsia, gestational diabetes, intrahepatic cholestasis of pregnancy, or substance abuse during pregnancy; (4) antibiotic use within 4 weeks prior to enrollment; (5) diagnosis of conditions potentially affecting growth and development within the previous month, including but not limited to pneumonia, severe gastrointestinal disorders (diarrhea, constipation, milk protein allergy), malnutrition, gastrointestinal surgery, neurological conditions (severe congenital heart disease, epilepsy, cerebral palsy, intellectual disability), hereditary metabolic disorders, chromosomal abnormalities, or genetic syndromes; (6) significant primary diseases affecting major organ systems (cardiac, hepatic, renal, hematopoietic); (7) participation in other clinical trials or use of investigational drugs within 3 months prior to screening; (8) consumption of probiotic supplements within 1 month before enrollment (or since birth for infants under 1 month of age); (9) previous use of immunosuppressive medications (glucocorticoids, immunosuppressants); (10) known allergy to any probiotic product components; (11) malnutrition requiring hospitalization; or (12) any other condition deemed by investigators to potentially compromise safety, efficacy assessment, or protocol adherence.

The sample size was determined based on the primary outcome of eczema incidence at 6 months. Referring to a previous randomized controlled trial investigating probiotic intervention for eczema prevention in infants (16), which reported an eczema incidence of 4.2% in the probiotic group compared to 11.5% in the control group, a total of 300 participants (150 per group) would provide 80% power to detect this difference at a two-sided significance level of α = 0.05. To account for an anticipated 20% attrition rate over the study period, the final sample size was increased to 360 participants, with 180 individuals allocated to each group.

The study was conducted as a multi-center trial in Chengdu (Chengdu Women & Children's Central Hospital), Chongzhou, Xindu, and Baoxing County, all located in Sichuan Province, China. These sites serve as major pediatric healthcare centers and collectively represent a typical urban-suburban population in southwestern China. The regional prevalence of childhood eczema and recurrent respiratory tract infections (20%−30%) is comparable to national epidemiological data reported for Chinese children, indicating that the study population is broadly representative of national pediatric patterns (17).

### 2.3 Intervention

Participants received one sachet daily where the probiotic group contained *B. lactis* BLa80 (5 × 10^9^ CFU per 2 g per day) with maltodextrin as excipient, while the placebo group contained identical sachets with excipient only. Parents were instructed to dissolve the sachet in lukewarm water or milk prior to ingestion. Compliance was monitored through daily logs and sachet counts, and participants with < 80% adherence were excluded from per-protocol analyses.

### 2.4 Clinical assessments and questionnaires

Three standardized instruments were employed: (i) baseline demographic questionnaire, (ii) serial anthropometric assessments, and (iii) structured clinical symptom diary.

Baseline data included age, sex, gestational age, birth mode, and family allergy history. Growth parameters (weight, length/height, and head circumference) were measured at baseline (Day 0) and at 180 days according to the WHO Child Growth Standards (2006). Measurements were taken in triplicate at each visit by trained personnel using calibrated equipment to minimize measurement variability.

Symptom assessment was conducted where parents recorded daily symptoms related to respiratory, gastrointestinal, and allergic manifestations, following standardized criteria used in pediatric outpatient monitoring. Clinical staff reviewed and verified symptom diaries at each visit.

### 2.5 Stool sample collection

Fecal samples (~5 g) were collected at Day 0 and Day 180 using sterile containers, stored at 4 °C, transported to the central laboratory within 72 h, and frozen at −80 °C until analysis. Samples were anonymized and labeled with study codes to maintain blinding.

### 2.6 DNA extraction and microbiota analyses

Total genomic DNA was extracted from fecal samples using the CTAB/SDS method combined with the QIAamp Fast DNA Fecal Mini Kit (Qiagen, Valencia, CA, USA) following the manufacturer's instructions. The bacterial 16S rRNA gene V3-V4 region was amplified using the TransGen AP221-02 Kit (TransGen, Beijing, China) with primers 341F and 806R. PCR products were purified, quantified, and sequenced (2 × 250 bp paired-end) on the Illumina MiSeq platform (Illumina, San Diego, CA, USA).

Raw reads were quality-filtered and merged using UPARSE v7.0.1001 (Robert C. Edgar/drive5, Tiburon, CA, USA), and chimeric sequences were removed. Operational taxonomic units (OTUs) were clustered at 97% sequence similarity, and representative sequences for each OTU were taxonomically classified using the RDP Classifier version 2.2 (Ribosomal Database Project, Center for Microbial Ecology, Michigan State University, East Lansing, MI, USA). The resulting OTU table was imported into QIIME v1.9.1 (QIIME Development Team, Boulder, CO, USA) for downstream diversity analyses. Functional predictions based on 16S rRNA gene profiles were performed using PICRUSt2 v2.6.0 (PICRUSt2 Development Team, Halifax, NS, Canada/Boston, MA, USA), with KEGG pathway mapping to infer the potential metabolic functions of the gut microbiota.

### 2.7 Differential taxonomy and pathway analysis

Microbial differential abundance was identified using LEfSe (α = 0.05, LDA ≥ 2.0). Functional pathway prediction was conducted using PICRUSt2 based on KEGG orthologs, and group comparisons were visualized using STAMP v2.1 (Beiko Lab, Dalhousie University, Halifax, NS, Canada). Microbial data integration and harmonization were carried out using Gi-MAPS (DiproX, Shanghai, China).

### 2.8 Immune biochemical indicators

All biochemical indicators of stool were assessed using a human protein-specific enzyme-linked immunosorbent assay (ELISA) kit (Shanghai Enzyme Linked Biotechnology Co., Ltd., Shanghai, China) in accordance with the manufacturer's instructions. Protein concentrations were calculated from a standard curve generated with known respective protein standards and expressed in the units indicated for each biomarker.

### 2.9 Statistical analysis

All statistical analyses were performed in R (version 4.3.2; R Core Team, R Foundation for Statistical Computing, Vienna, Austria). Alpha diversity (Chao1, Shannon, Simpson, Fisher) was calculated using the *vegan* package. Beta diversity was assessed by Bray–Curtis dissimilarity with PERMANOVA (999 permutations). Continuous variables were analyzed using Mann–Whitney *U*-tests and categorical data with χ^2^ tests or mid-p exact method.

To more rigorously evaluate intervention effects and account for potential confounding, multivariable regression models were constructed for key clinical outcomes. For binary disease outcomes (e.g., eczema, upper respiratory tract infection), multivariable logistic regression models were fitted including treatment group as the primary exposure and adjusting for pre-specified potential confounders. These confounders were selected *a priori* based on clinical relevance and baseline imbalance and included delivery mode (vaginal vs. cesarean), household registration (urban vs. rural), age at enrollment (continuous), sex, maternal education level (categorized as primary/junior/high school/college/university), and monthly household income per capita.

Logistic regression models were fitted using a logit link function, with treatment group as a fixed effect and demographic variables included as covariates. Generalized linear models for symptom frequency outcomes were specified with appropriate link functions depending on data distribution.

For symptom frequency outcomes expressed as incident rate per person per 100 intervention days, generalized linear models with appropriate link functions were applied.

To examine whether treatment effects differed across demographic strata, interaction terms (Group × Confounder) were introduced into the models. The statistical significance of interaction terms was assessed using Wald tests. Where significant interactions were identified, stratified analyses were performed to aid interpretation.

Longitudinal microbiota diversity indices and immune biomarkers were analyzed using Group × Phase interaction models, incorporating treatment group, time (phase), and their interaction as fixed effects, with participant included as a random effect where appropriate. No additional demographic covariates were included in these longitudinal microbiome models. *p* < 0.05 was considered statistically significant. No formal adjustment for multiple comparisons was applied for exploratory interaction analyses; therefore, these findings should be interpreted cautiously.

To evaluate potential effect modification, interaction terms between treatment group and key demographic variables were tested in multivariable models. Specifically, Group × Sex, Group × Maternal Education Level, Group × Household Registration (urban vs. rural), Group × Delivery Mode, and Group × Household Income were included. Interaction coefficients were assessed using Wald tests. A summary of interaction analyses for the primary clinical outcomes is provided in Supplementary Table S3.

To explore the ecological relevance of bifidobacterial dynamics, exploratory association analyses were conducted between the relative abundance of *Bifidobacterium* and primary clinical outcomes (eczema and upper respiratory tract infection). Multivariable regression models adjusting for demographic covariates were applied. Associations between *Bifidobacterium* abundance and fecal immune biomarkers (sIgA, calprotectin, and hBD-2) were further evaluated using Spearman correlation analysis. Results of these exploratory analyses are summarized in Supplementary Table S4.

### 2.10 Transparency statement

To promote transparency and reproducibility, the complete analysis pipeline, anonymized data structure, and code metadata are documented in the Supplementary materials and can be accessed upon reasonable request. All laboratory and computational protocols followed standardized procedures to ensure methodological consistency and reproducibility.

## 3 Results

### 3.1 Participant flow and baseline characteristics

**Figure 1:**
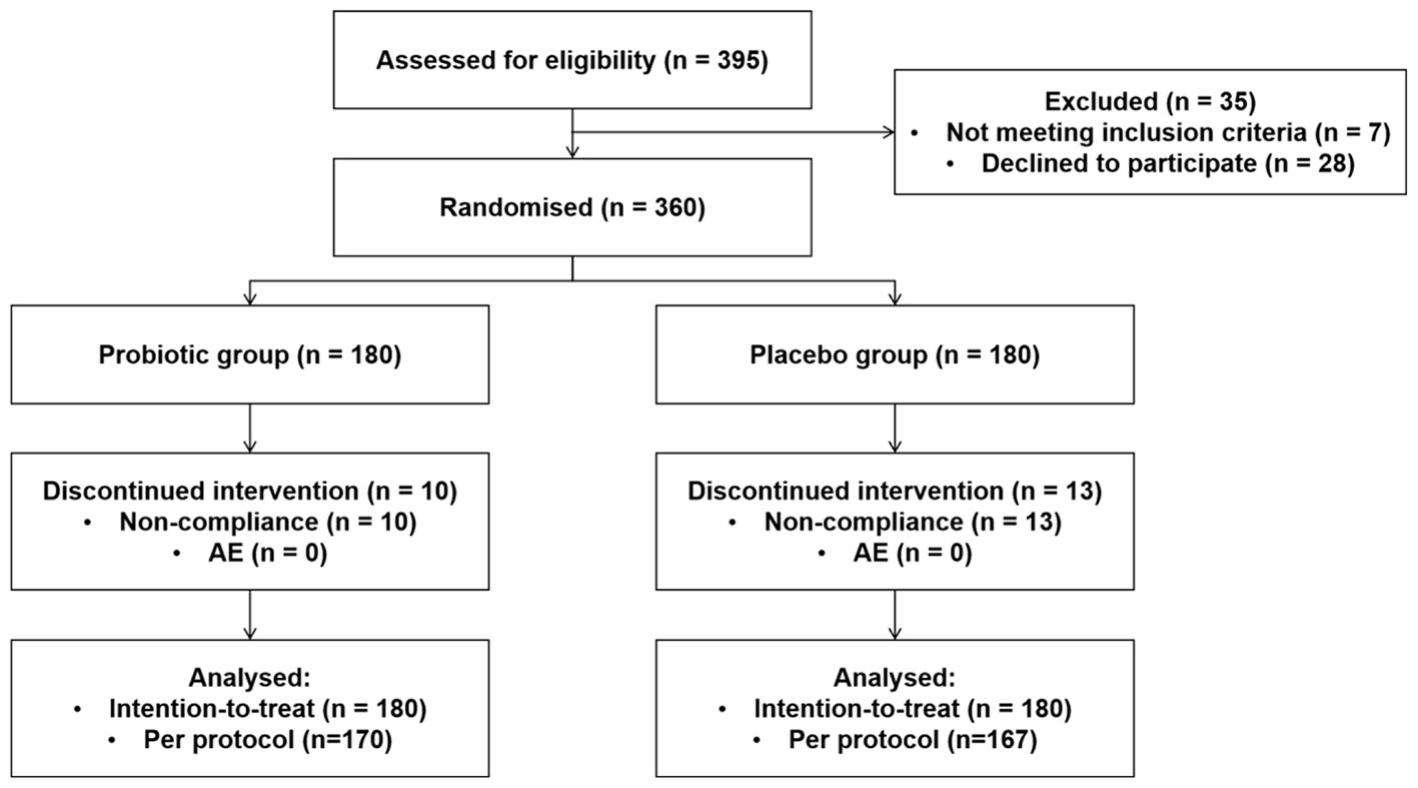
CONSORT flow of participant screening, randomization, allocation, and analysis inclusion. Of 395 screened infants and young children, 360 were randomized and 337 included in final analysis (170 probiotic, 167 placebo). No withdrawal or adverse events were reported, indicating good overall safety and compliance. CONSORT, consolidated standards of reporting trials.

Of the 395 infants and young children screened, 360 met eligibility criteria and were randomized into both groups (*n* = 180 per group; Figure 1). Twenty-three participants (probiotic *n* = 10, placebo *n* = 13) were excluded due to incomplete sampling such as questionnaires and/or fecal samples, yielding a final per protocol dataset of 337 (probiotic *n* = 170, placebo *n* = 167). Baseline demographic and clinical characteristics were well-balanced between groups (Table 1). No significant differences were observed in sex distribution, gestational age, birth weight, delivery mode, or family history of allergic disease (all *p* > 0.05), confirming successful randomization and group comparability.

**Table 1:** Baseline demographic and clinical characteristics of participants in the probiotic and placebo groups.

Variable	Probiotic	Placebo	*p-*value
Samples	170	167	—
Gender			
Male	95	90	0.811
Female	75	77	
Household registration			
Urban	122	122	0.702
Rural	48	45	
Ethnicity			
Han	169	166	0.972
Non-Han	1	1	
Delivery method			
Vaginal delivery	68	55	0.180
Cesarean section	102	112	
Age at enrollment (months)	14.52 ± 9.90	12.59 ± 9.21	0.095
Gestational week	38.65 ± 1.74	38.60 ± 1.55	0.802
Previous allergy history			
None	3	2	1.000
Present	167	165	
Family history of genetic diseases			
None	1	0	1.000
Present	169	167	
Length at enrollment	75.32 ± 11.08	72.92 ± 11.39	0.078
Weight at enrollment	9.37 ± 2.50	8.95 ± 2.67	0.208
Head circumference at enrollment	44.70 ± 3.33	44.09 ± 3.67	0.187
Mother's education level			
Primary school or below	0	2	0.745
Junior high school	27	24	
High school/technical secondary school	43	47	
Junior college/vocational college	52	47	
University or above	48	47	
Monthly household income per capita			
< 4,000	0	1	0.023
4,001–5,000	4	19	
5,001–6,000	13	18	
>6,000	153	129	

Data are presented as mean ± SD or n. There were no significant differences between groups for sex, gestational age, delivery method, birth weight, or family history of allergic diseases, confirming successful randomization and comparability before intervention. Statistical comparisons were conducted using the Mann-Whitney U-test or χ^2^ test. SD, standard deviation; *p* < 0.05 considered significant.

### 3.2 Clinical outcomes

#### 3.2.1 Safety and growth

The probiotic intervention demonstrated an excellent safety profile, with no serious adverse events reported. Growth indicators expressed as WHO *Z*-scores, including height-for-age, weight-for-age, and head circumference-for-age, showed appropriate developmental progression in both groups over the 180-day intervention period. No significant differences were detected between probiotic and placebo groups for any growth trajectory (all *p* > 0.05; Table 2), indicating that daily *B. lactis* BLa80 supplementation did not adversely affect normal pediatric growth and development.

**Table 2:** Growth and development indicators at baseline and day 180 in infants and young children.

Description	Day 0			Day 180			Difference day-0 and day-180		
	Probiotic *n* = 170	Placebo *n* = 167	*p*-value	Probiotic *n* = 170	Placebo *n* = 167	*p*-value	Probiotic *n* = 170	Placebo *n* = 167	*p*-value
Height-for-age	0.29 ± 1.51	0.23 ± 0.99	0.896	−0.84 ± 1.22	−0.85 ± 1.06	0.685	−1.13 ± 0.78	−1.07 ± 0.79	0.880
Weight-for-age	0.16 ± 1.24	0.24 ± 0.93	0.376	−0.40 ± 0.97	−0.25 ± 0.92	0.173	−0.56 ± 0.62	−0.49 ± 0.63	0.668
Head circumference-for-age	0.22 ± 1.23	0.25 ± 0.91	0.488	−0.25 ± 1.23	−0.29 ± 1.07	0.954	−0.47 ± 0.87	−0.55 ± 0.81	0.425

Data are presented as mean ± SD of WHO growth standard Z-scores for height-for-age, weight-for-age, and head circumference-for-age. No significant differences were detected between probiotic and placebo groups at baseline, at day 180, or for the change from baseline. Statistical comparisons were conducted using the Mann-Whitney U-test. SD, standard deviation; *p* < 0.05 considered significant.

#### 3.2.2 Symptom burden and incidence

Analysis of symptom burden, summarized as both incident rate per person per 100 intervention days and proportion of affected participants, revealed multiple significant differences between groups (Table 3). The probiotic group showed consistently lower frequency and occurrence of several symptoms compared with the placebo group. The probiotic group had markedly fewer respiratory symptoms such as nasal congestion (frequency 1.136 vs. 2.095%, *p* < 0.001; affected participants 38.8 vs. 55.7%, *p* = 0.002), cough (affected participants 65.3 vs. 82.6%, *p* < 0.001), nasal discharge (frequency 2.904 vs. 3.314%, *p* = 0.005; affected participants 60.6 vs. 75.4%, *p* = 0.004) and milk aspiration (frequency 0.210 vs. 0.448%, *p* < 0.001; affected participants 12.9 vs. 25.7%, *p* = 0.003). For gastrointestinal symptoms, significant reductions were observed in vomiting (frequency 0.175 vs. 0.365%, *p* < 0.001; affected participants 14.1 vs. 29.9%, *p* < 0.001), food refusal (frequency 0.353 vs. 0.721%, *p* < 0.001; affected participants 19.4 vs. 31.7%, *p* = 0.048) and decreased appetite (frequency 0.360 vs. 0.683%, *p* < 0.001; affected participants 18.2 vs. 32.9%, *p* = 0.002). Systemic symptoms were also less frequent, including pyrexia (frequency 0.835 vs. 1.083%, *p* = 0.002), irritability (frequency 0.349 vs. 0.629%, *p* < 0.001; affected participants 15.9 vs. 28.7%, *p* = 0.023) and eczematous eruption (frequency 1.754 vs. 7.140%, *p* < 0.001; affected participants 27.6 vs. 69.5%, *p* < 0.001). No significant differences were found for other symptoms including flatulence, hard stools, or reflux (all *p* > 0.05).

**Table 3:** Incidence and frequency of respiratory, gastrointestinal, and systemic symptoms during the 180-day intervention period.

Symptoms	Probiotic *n* = 170		Placebo *n* = 167		*p*-Value^a^	Number of people reported having each symptom over 180 days				
	Total occurrence days	The incident rate per person per 100 intervention days (%)	Total occurrence days	The incident rate per person per 100 intervention days (%)		Probiotic *n* = 170	Placebo *n* = 167	RR	95% CI	*p*-value^b^
Respiratory symptoms										
Nasal congestion	325	1.136	660	2.095	< 0.001	66 (38.8)	93 (55.7)	0.697	(0.553, 0.879)	0.002
Cough	1,169	4.085	1,302	4.133	0.789	111 (65.3)	138 (82.6)	0.790	(0.694, 0.900)	< 0.001
Nasal discharge	831	2.904	1,044	3.314	0.005	103 (60.6)	126 (75.4)	0.803	(0.692, 0.932)	0.004
Milk aspiration	60	0.210	141	0.448	< 0.001	22 (12.9)	43 (25.7)	0.503	(0.315, 0.802)	0.003
Gastrointestinal symptoms										
Flatulence	53	0.185	82	0.260	0.064	16 (9.4)	26 (15.6)	0.605	(0.337, 1.085)	0.088
Hard stools	176	0.615	194	0.616	1.000	31 (18.2)	45 (26.9)	0.677	(0.452, 1.014)	0.056
Reflux	27	0.094	37	0.117	0.458	11 (6.5)	16 (9.6)	0.675	(0.323, 1.412)	0.294
Milk curds	143	0.5	119	0.378	0.028	22 (12.9)	23 (13.8)	0.940	(0.545, 1.619)	0.823
Vomiting	50	0.175	115	0.365	< 0.001	24 (14.1)	50 (29.9)	0.472	(0.304, 0.730)	< 0.001
Food refusal	101	0.353	227	0.721	< 0.001	33 (19.4)	53 (31.7)	0.612	(0.419, 0.893)	0.048
Decreased appetite	103	0.360	215	0.683	< 0.001	31 (18.2)	55 (32.9)	0.554	(0.377, 0.814)	0.002
Other symptoms										
Pyrexia	239	0.835	341	1.083	0.002	76 (44.7)	93 (55.7)	0.899	(0.726, 1.114)	0.332
Irritability	100	0.349	198	0.629	< 0.001	27 (15.9)	48 (28.7)	0.619	(0.407, 0.942)	0.023
Eczematous eruption	502	1.754	2,249	7.140	< 0.001	47 (27.6)	116 (69.5)	0.446	(0.343, 0.580)	< 0.001

Data are expressed as total occurrence days, incident rate per person per 100 intervention days (%), and number [n (%)] of participants who experienced each symptom at least once during the 180-day intervention. The probiotic group showed significantly lower incidence of nasal congestion, nasal discharge, milk aspiration, vomiting, food refusal, decreased appetite, pyrexia, irritability, and eczematous eruption compared with the placebo group.

^a^p-values were calculated using the χ^2^ test to compare incident rates between groups.

^b^p-values were calculated from RR and 95% CIs using the mid-p exact method. RR, risk ratio; CI, confidence interval. *p* < 0.05 considered significant.

Disease incidence analysis showed significantly lower rates of eczema (27.6 vs. 69.5%, RR: 0.398, 95% CI: 0.306–0.518, *p* < 0.001), upper respiratory tract infections (19.4 vs. 42.5%, RR: 0.457, 95% CI: 0.321–0.650, *p* < 0.001), and tracheitis (10.0 vs. 19.2%, RR: 0.522, 95% CI: 0.302–0.903, *p* = 0.017) in the probiotic group compared to placebo (Table 4). No significant differences were detected for pneumonia, diarrhea, constipation, dyspepsia, or colic incidence between groups (all *p* > 0.05).

**Table 4:** Disease incidence and relative risk during the 180-day intervention period.

Disease	Probiotic *n* = 170	Placebo *n* = 167	RR	95% CI	*p*-value
Eczema	47 (27.6)	116 (69.5)	0.398	(0.306, 0.518)	< 0.001
Upper respiratory tract infection	33 (19.4)	71 (42.5)	0.457	(0.321, 0.650)	< 0.001
Tracheitis	17 (10.0)	32 (19.2)	0.522	(0.302, 0.903)	0.017
Pneumonia	15 (8.8)	23 (13.8)	0.641	(0.347, 1.184)	0.152
Diarrhea	39 (22.9)	28 (16.8)	1.368	(0.885, 2.116)	0.152
Constipation	4 (2.4)	9 (5.4)	0.437	(0.137, 1.390)	0.149
Dyspepsia	11 (6.5)	16 (9.6)	0.675	(0.323, 1.412)	0.294
Colic	8 (4.7)	13 (7.8)	0.605	(0.257, 1.421)	0.243

Data are presented as the number of affected participants [n (%)]. The probiotic group experienced fewer cases of eczema, upper respiratory tract infection and trachetis compared with the placebo group. p-values were derived from RR and 95% CIs using the mid-p exact method. RR, risk ratio; CI, confidence interval. *p* < 0.05 considered significant.

Multivariable regression analyses adjusting for delivery mode, household registration, age at enrollment, sex, maternal education level, and monthly household income confirmed that the protective effects of BLa80 supplementation on eczema and upper respiratory tract infection remained statistically significant.

Interaction analyses were subsequently performed to determine whether treatment effects differed across demographic subgroups. No significant interaction effects were observed between treatment group and sex, maternal education level, or household registration for the primary outcomes of eczema and upper respiratory tract infection (all *p* for interaction > 0.05), indicating consistent probiotic effects across these strata. Detailed interaction coefficients and corresponding *p*-values are summarized in Supplementary Table S3.

### 3.3 Gut microbiota composition

#### 3.3.1 Diversity indices

To evaluate whether temporal changes differed between groups, analyses focused on Group × Phase interaction models, with interpretation centered on between-group differences in trajectories over time rather than within-group pre/post comparisons.

**Figure 2:**
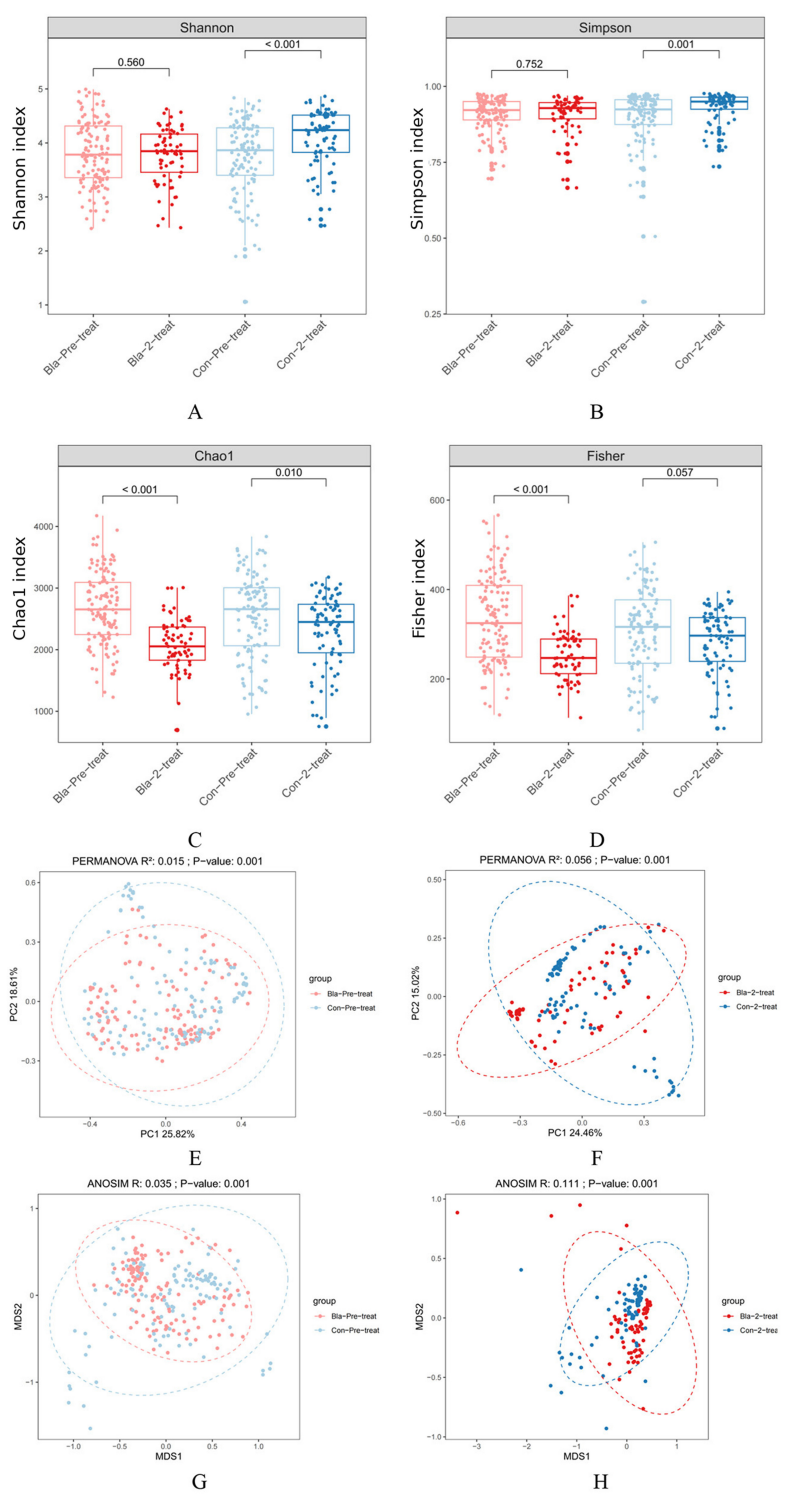
Gut microbiota diversity before and after probiotic intervention. **(A–D)** Changes in alpha diversity indices including **(A)** Shannon, **(B)** Simpson, **(C)** Chao1, and **(D)** Fisher across baseline (Pre-treat) and post-intervention (two-treat) samples in the probiotic and placebo groups. Shannon and Simpson indices reflect community evenness, while Chao1 and Fisher indicate richness. The probiotic group showed significant decreases in richness indices (Chao1, Fisher) after 180 days, whereas the placebo group exhibited significant increases in evenness indices (Shannon, Simpson). Boxes represent interquartile ranges, horizontal lines indicate medians, and dots represent individual samples. **(E–H)** Beta diversity analyses based on Bray-Curtis dissimilarity assessed by PCoA **(E, F)** and NMDS **(G, H)**. Significant compositional differences were observed between the probiotic and placebo groups both at baseline and after intervention. Increased *R*^2^ and *R* values at Day 180 indicate a higher degree of community differentiation following probiotic supplementation. Ellipses represent 95% confidence intervals for group clustering. PCoA, principal coordinates analysis; NMDS, non-metric multidimensional scaling. *p* < 0.05 considered significant.

Distinct structural changes of gut microbiota diversity between intervention groups were observed over time (Figure 2). Alpha diversity metrics demonstrated contrasting trajectories where the probiotic group exhibited reduced richness, as evidenced by significant decreases in Chao1 and Fisher indices (*p* < 0.001), while maintaining stable community evenness (Shannon and Simpson indices, *p* > 0.05). Conversely, the placebo group showed significant increases in both Shannon (*p* < 0.001) and Simpson (*p* = 0.001) indices over time.

Group × Phase interaction models demonstrated that temporal changes in α-diversity differed significantly between groups (Supplementary Table S1). Significant interaction effects were observed for Shannon (β = 0.46, *p* < 0.001) and Simpson indices (β = 0.05, *p* = 0.003), indicating that community evenness evolved differently in the probiotic group compared with placebo. Similarly, richness metrics demonstrated significant group-specific trajectories, with positive interactions for Chao1 (β = 333.92, *p* = 0.006) and Fisher indices (β = 56.56, *p* = 0.001). These findings indicate that the divergence in microbial diversity patterns observed over the 180-day intervention cannot be attributed solely to age-related maturation, and is consistent with intervention-associated ecological remodeling. These significant interaction effects confirm that observed differences in richness and evenness reflect differential temporal trajectories between groups rather than simple within-group changes.

Beta diversity analysis further highlighted the profound impact of BLa80 supplementation on microbial community structure. Bray-Curtis dissimilarity metrics revealed significant compositional separation between groups at both baseline (PERMANOVA *R*^2^ = 0.015, *p* = 0.001; ANOSIM R = 0.035, *p* = 0.001) and study completion (PERMANOVA *R*^2^ = 0.056, *p* = 0.001; ANOSIM R = 0.111, *p* = 0.001). The substantial increase in both *R*^2^ and *R* values at Day 180 demonstrates that probiotic supplementation amplified pre-existing compositional differences, resulting in a distinctly diverged microbial ecosystem by the endpoint of the intervention.

#### 3.3.2 Microbial taxonomic shifts

**Figure 3:**
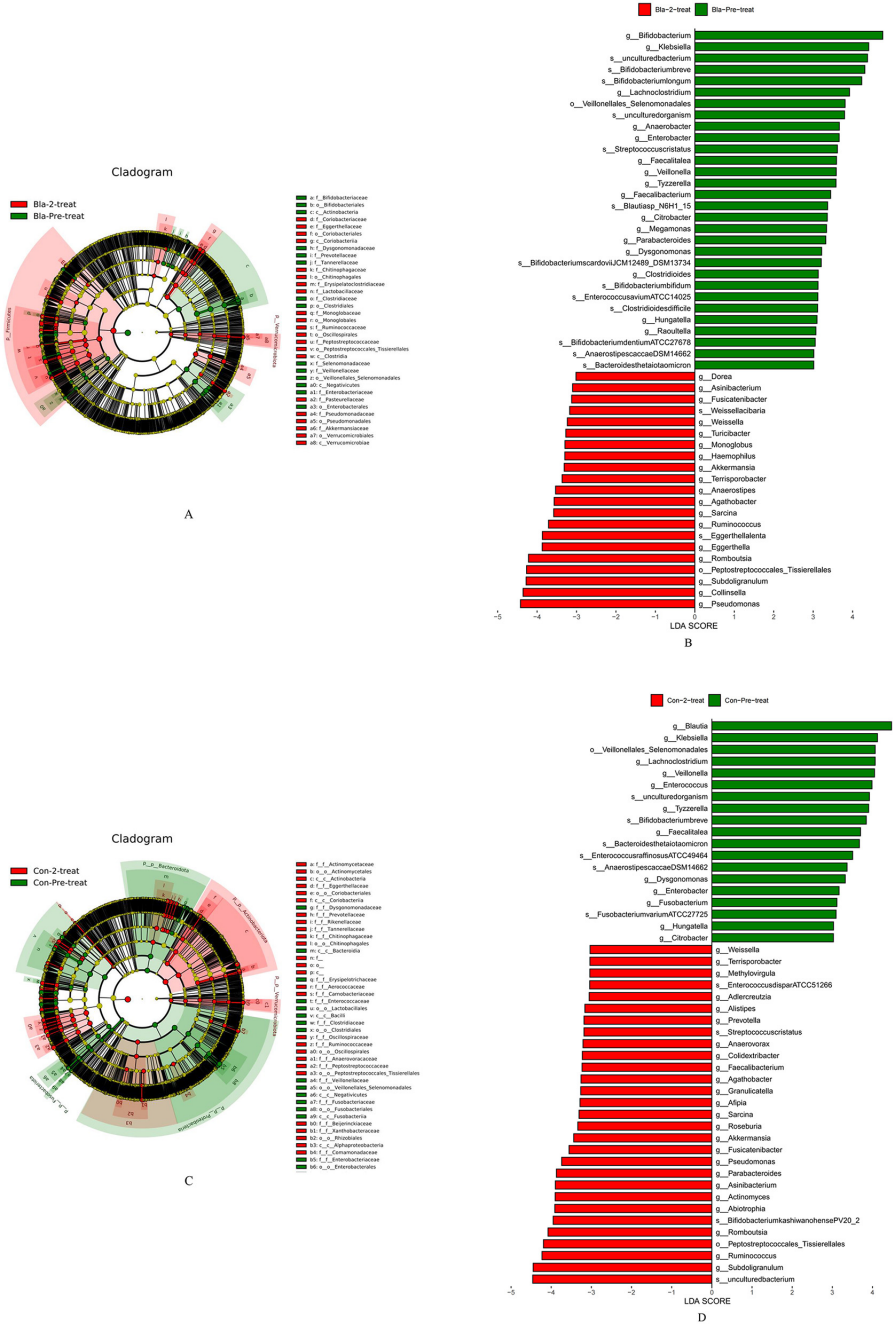
LEfSe-based identification of differential gut microbiota between baseline and post-intervention within each group. **(A, B)** Differentially enriched taxa in the probiotic group between baseline (Pre-treat, green) and Day 180 (two-treat, red), displayed as a cladogram **(A)** and LDA score plot **(B)**. The baseline microbiota was dominated by *Bifidobacterium* and *Klebsiella*, whereas post-intervention samples were enriched in *Dorea, Fusicatenibacter, Weissella, Akkermansia*, and *Ruminococcus*. **(C, D)** Differentially enriched taxa in the placebo group between baseline (Pre-treat, green) and Day 180 (two-treat, red). The baseline community was characterized by *Blautia, Klebsiella*, and *Lachnoclostridium*, while post-intervention enrichment was observed for *Weissella, Prevotella*, and *Faecalibacterium*. Probiotic intake selectively promoted beneficial genera and prevented enrichment of opportunistic taxa associated with inflammation. Cladograms depict phylogenetic relationships of significantly different taxa, and LDA bar plots indicate effect size and direction of enrichment. LDA threshold ≥2.0, *p* < 0.05. LDA, linear discriminant analysis; LEfSe, linear discriminant analysis effect size.

Analysis via LEfSe revealed significant alterations in microbial taxa abundance between baseline and day 180 in both groups (Figure 3). In the probiotic group, the baseline microbiota was characterized by significant abundance of *Bifidobacterium* (genus and species levels including *B. breve, B. longum, B. scardovii, B. bifidum*, and *B. dentium*), alongside *Klebsiella, Lachnoclostridium*, and *Clostridioides difficile*. Following intervention, the probiotic group demonstrated a significant shift toward a more diverse community including *Dorea, Fusicatenibacter, Weissella* (particularly *W. cibaria*), *Akkermansia, Ruminococcus*, and *Eggerthella lenta*.

The placebo group at baseline was characterized by *Blautia, Klebsiella, Lachnoclostridium, Veillonella*, and multiple *Enterococcus* species (*E. raffinosus*). Post-intervention, the placebo group showed significant expansion of *Weissella, Terrisporobacter, Prevotella, Faecalibacterium, Agathobacter*, and multiple *Ruminococcus* and *Subdoligranulum* species.

Between-group comparisons using LEfSe further supported these observations (Supplementary Figure 1). At baseline, the probiotic group was enriched with *Bifidobacterium* and related species, along with genera such as *Collinsella, Coprococcus*, and *Lactobacillus*, whereas the placebo group showed enrichment of taxa including *Akkermansia, Fusobacterium*, and *Pseudomonas*. At the end of the intervention, the probiotic group was characterized by enrichment of genera such as *Dorea, Blautia, Collinsella*, and *Romboutsia*, while the placebo group exhibited broader enrichment of taxa including *Bifidobacterium, Akkermansia, Prevotella, Ruminococcus*, and *Subdoligranulum*. At the species level, the probiotic group retained enrichment of select beneficial taxa such as *Bifidobacterium bifidum* and *Lactobacillus mucosae*, whereas the placebo group showed enrichment of species including *Bifidobacterium breve, B. longum*, and *Clostridioides difficile*.

Importantly, these between-group differences are consistent with the longitudinal within-group trends and do not alter the overall interpretation of the findings.

Analysis of microbial taxonomic shifts revealed that probiotic supplementation induced a specific and beneficial restructuring of the gut community, distinct from the changes observed in the placebo group. The intervention consistently promoted the establishment of beneficial genera, such as *Akkermansia* and *Fusicatenibacter*, while facilitating a marked transition from an initial *Bifidobacterium*-dominated state to a more diverse and stable post-intervention ecology. These findings suggest that the probiotic may influence gut microbial composition by orchestrating specific, significant, and potentially health-promoting alterations in the gut microbial community.

#### 3.3.3 Exploratory comparison of microbiota in children with and without eczema

To address potential differences in microbiota associated with clinical outcomes, comparisons were performed between children who developed eczema and those who remained eczema-free within each treatment group. Exploratory analyses were conducted to examine whether children who developed eczema exhibited distinct microbial patterns at Day 180. Across the full cohort, α-diversity metrics, including Shannon, Simpson, Chao1 and Fisher indices, were consistently higher in children with eczema compared with those without eczema (*p* < 0.001; Supplementary Figure 1). Notably, this pattern was evident within the BLa80 intervention group (*p* < 0.05), whereas the placebo group showed no clear or consistent separation, indicating that microbiota differences associated with eczema status were more pronounced within the intervention arm.

β-diversity analysis similarly demonstrated modest but statistically significant compositional separation between eczema and non-eczema samples (*p* < 0.05). Notably, this separation was more pronounced within the BLa80 group (*R*^2^ = 0.064) than within the placebo group (*R*^2^ = 0.035), suggesting that microbial community structure in the intervention arm was more coherent and allowed clearer differentiation of host phenotypes (Supplementary Figure 2).

### 3.4 Gut immune biomarkers

Group × Phase interaction models were applied to assess whether longitudinal changes in immune biomarkers differed between groups, and interpretation is based on between-group differences in temporal patterns rather than within-group changes alone.

**Figure 4:**
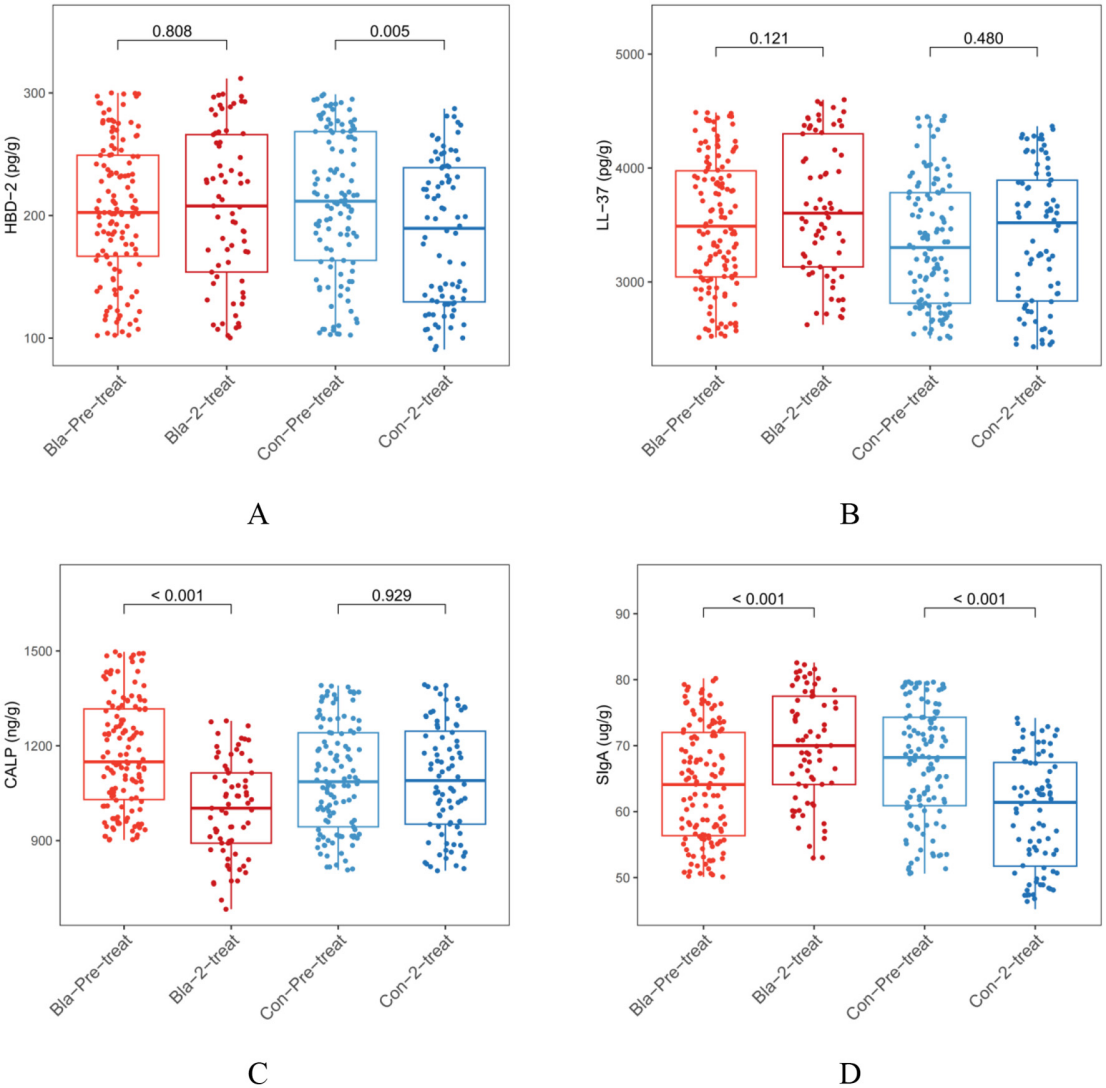
Changes in fecal immune biomarkers following 180 days of probiotic intervention. **(A)** human β-defensin-2 (hBD-2), **(B)** cathelicidin (LL-37), **(C)** Calprotectin (CALP), and **(D)** secretory immunoglobulin A (sIgA) concentrations were measured in fecal samples at baseline (Pre-treat) and after 180 days (two-treat) in the probiotic and placebo groups. Probiotic supplementation significantly reduced CALP and increased sIgA, while maintaining stable hBD-2 levels. In contrast, the placebo group showed decreased sIgA and hBD-2, with no changes in CALP. Data are presented as boxplots showing medians and interquartile ranges; dots represent individual values. Statistical comparisons were conducted using the Mann-Whitney *U*-test. *p* < 0.05 considered significant.

Analysis of fecal immune markers revealed distinct immunomodulatory effects between groups (Figure 4). At baseline, the probiotic group exhibited a higher gut concentration of calprotectin (CALP) compared to the placebo group (*p* = 0.001), but this trend reversed by the end of the intervention, with significantly lower CALP levels observed in the probiotic group (*p* = 0.001; Supplementary Figure 2). Consistently, CALP trajectories differed between groups over time, with a greater reduction observed in the probiotic group (Figure 4C).

Concentration of human Beta-Defensin 2 (hBD-2) was comparable between groups at baseline, but became significantly higher in the probiotic group at the end of the intervention (*p* = 0.029; Supplementary Figure 2). Longitudinally, hBD-2 remained stable in the probiotic group but decreased in the placebo group (*p* = 0.005; Figure 4A). For cathelicidin (LL-37), levels were consistently higher in the probiotic group than in the placebo group at both baseline and post-intervention timepoints (*p* < 0.05; Supplementary Figure 2), although no significant temporal changes were observed within either group (Figure 4B). Secretory immunoglobulin A (sIgA) trajectories differed between groups over time, with the probiotic group showing an increase relative to the declining pattern observed in the placebo group (Supplementary Figure 2). In contrast, sIgA decreased over time in the placebo group (*p* < 0.001; Figure 4D). Collectively, these findings suggest that probiotic supplementation may sustain key innate immune defenses, enhance adaptive mucosal immunity, and reduce intestinal inflammation, in contrast to the placebo.

To determine whether the temporal trajectories of immune biomarkers differed between groups, Group × Phase interaction models were applied (Supplementary Table S2). CALP demonstrated a significant negative interaction (β = −182.32, *p* < 0.001), indicating a greater reduction in the probiotic group compared with placebo. For hBD-2, the positive interaction term (β = 28.06, *p* = 0.015) showed that the probiotic group maintained higher levels over time relative to placebo. sIgA exhibited the strongest treatment-related divergence, with a significant positive interaction (β = 13.31, *p* < 0.001), consistent with enhanced mucosal immunity. No significant interaction was observed for LL-37 (*p* = 0.332). These results confirm that changes in CALP, hBD-2, and sIgA over time cannot be explained solely by natural maturation, and that BLa80 supplementation produced distinct immunological trajectories.

Notably, baseline differences were observed in certain immune markers, with higher calprotectin and lower sIgA levels in the probiotic group at Day 0. Although randomization was performed, such imbalances may occur by chance in clinical trials. Therefore, interpretation of these markers focuses on differences in longitudinal trajectories and Group × Phase interaction effects rather than absolute between-group comparisons at individual time points.

### 3.5 Gut microbiota functional profiling

**Figure 5:**
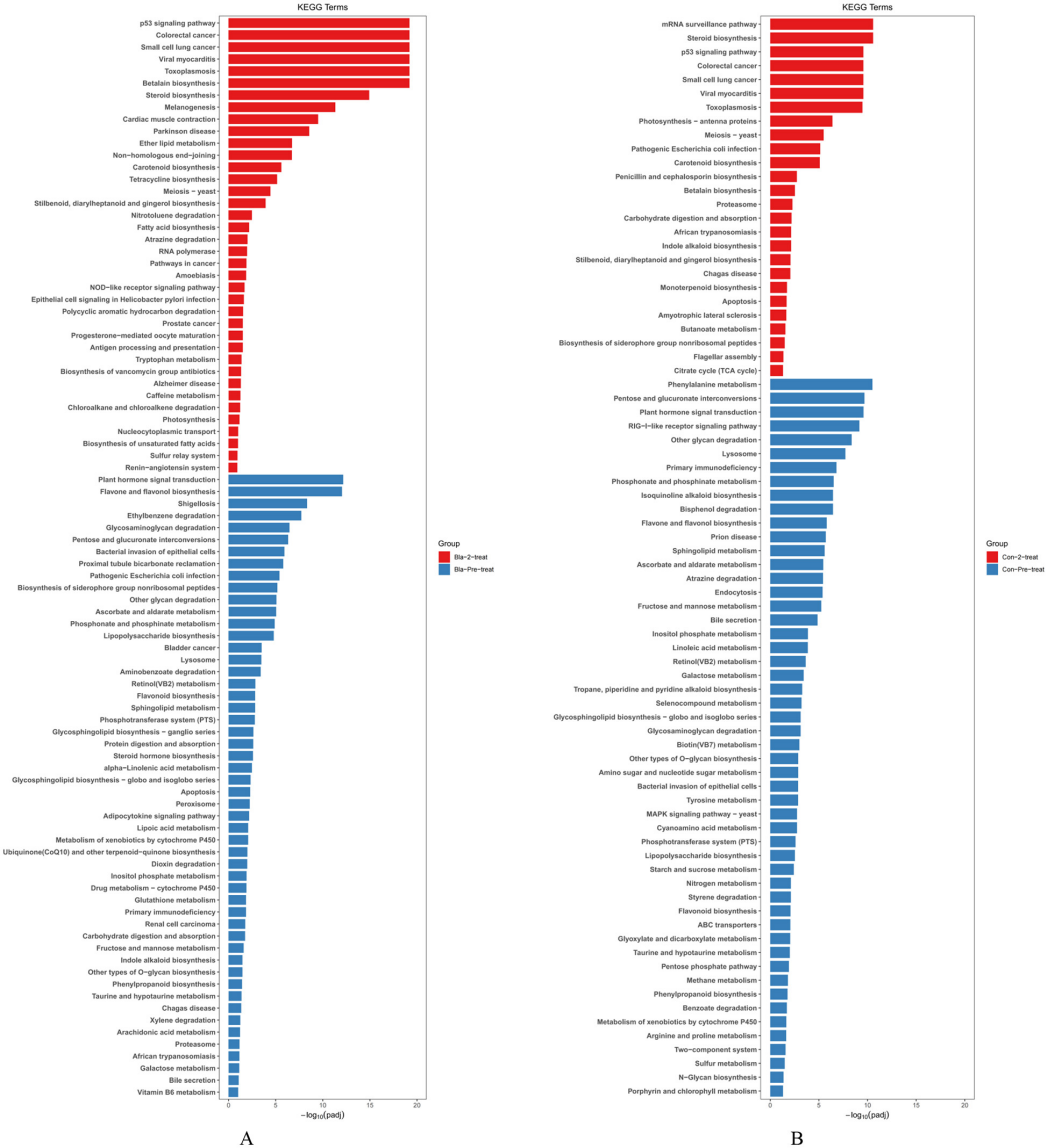
KEGG functional pathway enrichment following 180 days of intervention. **(A)** Enriched KEGG pathways in the probiotic group (Day 180 vs. baseline). **(B)** Enriched KEGG pathways in the placebo group (Day 180 vs. baseline). Red bars represent pathways upregulated after 180 days of intervention (two-treat), and blue bars represent pathways enriched at baseline (Pre-treat). Functional predictions were performed using PICRUSt2 based on 16S rRNA gene data, and KEGG pathway enrichment was visualized using STAMP. KEGG, kyoto encyclopedia of genes and genomes.

Functional analysis of the gut microbiota via KEGG pathway analysis revealed distinct patterns of pathway enrichment between the probiotic and placebo groups following the 180-day intervention (Figure 5). In the probiotic group, significant upregulation was observed across multiple metabolic pathways including ether lipid metabolism, fatty acid biosynthesis, and tryptophan metabolism (Figure 5A). The intervention group also demonstrated enhanced capacity for xenobiotic degradation, evidenced by upregulated pathways for nitrotoluene degradation, atrazine degradation, and polycyclic aromatic hydrocarbon degradation. Additionally, the probiotic group showed unique enrichment in pathways related to non-homologous end-joining and RNA polymerase under genetic information processing, along with NOD-like receptor signaling pathway and antigen processing and presentation in organismal systems.

In contrast, the placebo group exhibited upregulation of markedly different pathways, including apoptosis and flagellar assembly in cellular processes, and mRNA surveillance pathway in genetic information processing (Figure 5B). Disease-associated pathways showed distinct patterns, with the placebo group displaying enrichment for pathogenic Escherichia coli infection, African trypanosomiasis, and Chagas disease, while the probiotic group showed upregulation of Parkinson disease, prostate cancer, and Helicobacter pylori infection pathways. Metabolic pathways upregulated in the placebo group included penicillin and cephalosporin biosynthesis, indole alkaloid biosynthesis, and monoterpenoid biosynthesis.

Between-group comparisons further supported these functional differences (Supplementary Figure 3). At baseline, the probiotic group showed enrichment of pathways largely related to core metabolism and vitamin biosynthesis, including nicotinate and nicotinamide metabolism, vitamin B6 metabolism, folate biosynthesis, amino acid biosynthesis, and fatty acid degradation, alongside fundamental cellular processes such as ribosome function and DNA replication. In contrast, the placebo group at baseline was relatively enriched in pathways such as ether lipid metabolism, bile secretion, xenobiotic degradation (e.g., nitrotoluene and atrazine degradation), and disease-related pathways including Parkinson disease.

Following the intervention, the probiotic group exhibited strong enrichment of pathways consistent with enhanced metabolic versatility and immune interaction, including biotin metabolism, linoleic acid metabolism, amino acid metabolism, xenobiotic degradation pathways, and immune-related pathways such as NOD-like receptor signaling and antigen processing and presentation. In comparison, the placebo group showed enrichment of pathways associated with infection and disease (e.g., pathogenic *Escherichia coli* infection, Shigellosis), as well as metabolic pathways such as butanoate metabolism, glycosphingolipid biosynthesis, and continued enrichment of xenobiotic degradation and antibiotic biosynthesis pathways.

Importantly, these between-group differences are consistent with the longitudinal within-group findings and do not alter the overall interpretation.

**Figure 6:**
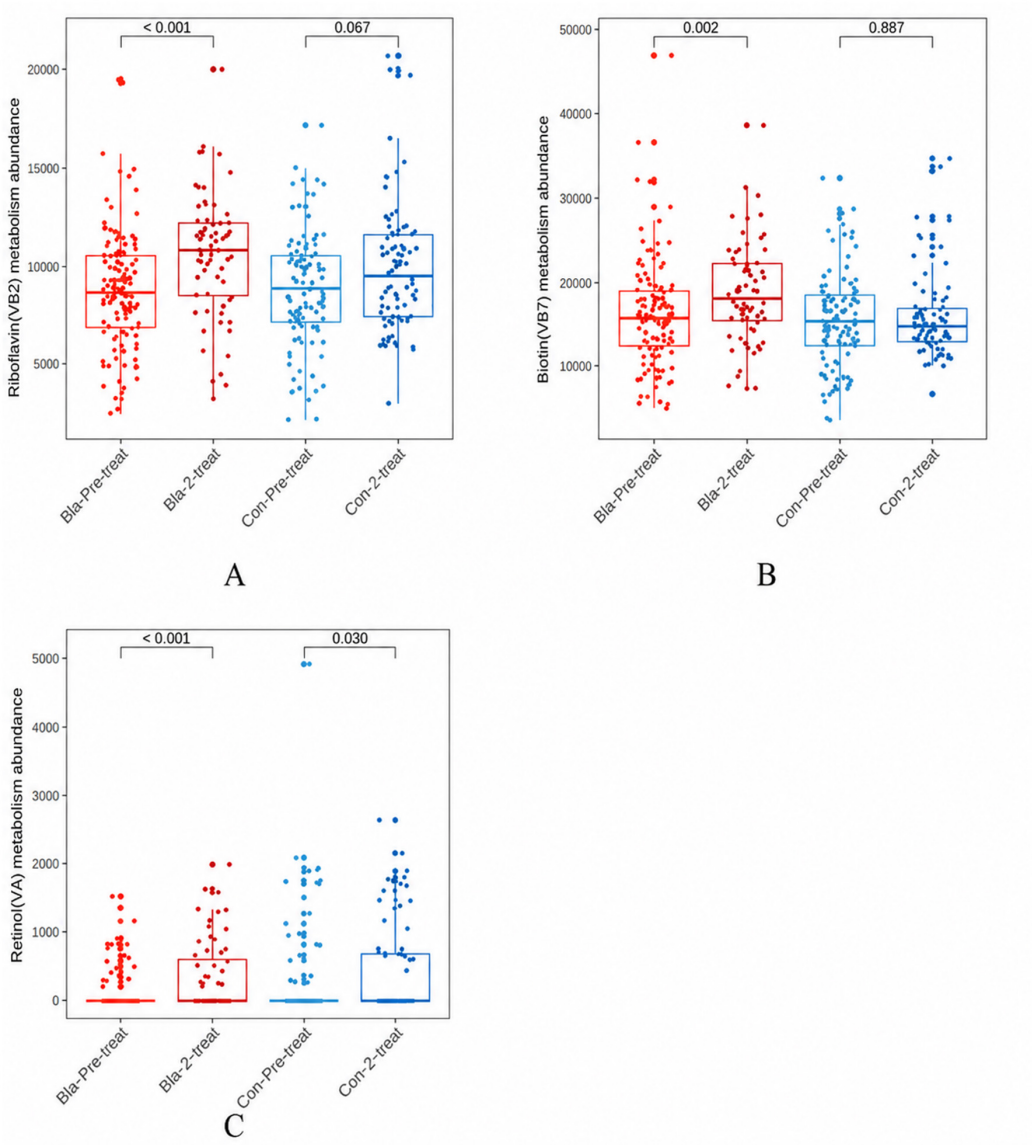
Alterations in vitamin-associated metabolic pathways predicted by KEGG before and after intervention. **(A)** riboflavin (vitamin B2) metabolism, **(B)** biotin (vitamin B7) metabolism, **(C)** retinol (vitamin A) metabolism. Comparisons were made between baseline (Pre-treat) and after 180 days of intervention (two-treat). Riboflavin and biotin pathways increased in the probiotic group with no significant change in the placebo group. Both groups showed increases in these vitamin biosynthesis pathways after 180 days, but the changes were more pronounced in the probiotic group. Data are presented as boxplots showing medians and interquartile ranges; dots represent individual values. Statistical comparisons were conducted using the Mann-Whitney *U*-test. *p* < 0.05 considered significant. KEGG, kyoto encyclopedia of genes and genomes.

The probiotic group also showed higher regulation of vitamin metabolism compared to the placebo group (Figure 6). This included riboflavin and biotin, where the probiotic group demonstrated upregulation over time (*p* < 0.05), while no significant changes were observed in the placebo group. Notably, regulation of biotin was comparable between groups at baseline but became significantly higher in the probiotic group at the end of the intervention (*p* < 0.001; Supplementary Figure 4). In contrast, regulation of riboflavin remained similar between groups at both baseline and post-intervention timepoints (Supplementary Figure 4), despite the observed within-group increase over time in the probiotic group.

The probiotic group also showed greater upregulation of retinol metabolism over time compared to the placebo (*p* < 0.001 vs. *p* = 0.030). However, between-group comparisons indicated that retinol regulation remained similar at both baseline and after the intervention (Supplementary Figure 4).

The distinct pathway enrichment patterns observed following the 180-day intervention demonstrate that probiotic supplementation induces a unique functional shift in the gut microbiome of children, distinct from placebo. The upregulation of critical metabolic pathways, including those for fatty acid biosynthesis, tryptophan metabolism, vitamin metabolism, and xenobiotic degradation, alongside enhanced activity in immune-regulatory pathways such as NOD-like receptor signaling and antigen presentation, suggests that the probiotic may confer benefits through modulation of the metabolic capacity and immune-interactive functions of the host's gut microbiota.

### 3.6 Association between *Bifidobacterium* abundance and clinical/immune outcomes

Exploratory analyses were performed to assess whether the relative abundance of *Bifidobacterium* was associated with primary clinical outcomes and immune biomarkers.

Regression analyses adjusting for treatment group and demographic variables were conducted to evaluate associations with eczema and upper respiratory tract infection. In addition, Spearman correlation analyses were applied to examine relationships between *Bifidobacterium* abundance and fecal immune markers (sIgA, calprotectin, and hBD-2).

Other than eczema, all other analyses did not demonstrate strong evidence of effect modification by bifidobacterial abundance on clinical and immune outcomes (Supplementary Table S4). However, trends observed suggest that ecological variation in bifidobacteria may contribute to immune modulation, warranting further investigation using strain-level approaches.

## 4 Discussion

This randomized controlled trial provides comprehensive evidence that 180-day supplementation with *Bifidobacterium animalis* subsp. *lactis* BLa80 induces beneficial remodeling of the gut ecosystem in early childhood, resulting in significant clinical improvements across multiple domains of infant health. The integration of detailed symptom analysis with multi-omics profiling reveals patterns consistent with potential mechanisms through which this probiotic strain may exert its effects.

The comprehensive symptom analysis revealed striking differences between groups that extend beyond the primary outcomes of eczema and respiratory infections. The substantial reduction in feeding-related symptoms is particularly noteworthy. The significant decreases in milk aspiration, vomiting, and food refusal in the probiotic group suggest improved feeding tolerance and coordination. These improvements may be linked to the observed taxonomic shifts, particularly the reduction in *Klebsiella* and enrichment of *Fusicatenibacter*, as gut microbiota composition has been shown to influence gastroesophageal reflexes and feeding behavior through the gut-brain axis (18, 19). The parallel improvement in decreased appetite further supports enhanced feeding efficiency in the probiotic group.

The significant reduction in nasal congestion and nasal discharge, coupled with the decreased incidence of upper respiratory tract infections, demonstrates the probiotic's substantial impact on respiratory health. This observation is consistent with potential gut–lung axis interactions, supported by the enrichment of immune signaling pathway, antigen processing and presentation in the functional analysis, suggests enhanced mucosal immune activity (20, 21). The reduction in irritability may reflect both decreased discomfort from respiratory and gastrointestinal symptoms, as well as potential direct effects of microbial metabolites on neural signaling, possibly mediated through the observed enhancements in tryptophan metabolism (22, 23).

The substantial reduction in eczema incidence represents the most significant clinical finding, consistent with the observed taxonomic restructuring. The transition from an initial state dominated by *Bifidobacterium* species alongside potential pathogens like *Klebsiella* and *Clostridioides difficile* to a more diverse ecosystem enriched with *Akkermansia, Fusicatenibacter*, and *Ruminococcus* suggests ecological maturation toward a more stable and protective microbial community. Particularly noteworthy is the enrichment of *Akkermansia*, a mucin-degrader known to enhance gut barrier function (24), and Fusicatenibacter, a butyrate producer with anti-inflammatory properties (25). Concurrently, the reduction in *Klebsiella*, a genus associated with systemic inflammation and gut barrier disruption, provides a plausible microbial basis for the decreased eczema risk (26).

An important limitation of this study is that 16S rRNA gene sequencing cannot distinguish the administered BLa80 strain from endogenous *Bifidobacterium* species, and therefore strain-level detection of BLa80 was not possible. The use of 16S rRNA V3–V4 sequencing provides genus-level resolution and does not allow strain-specific identification (27). Therefore, the persistence or abundance of BLa80 in the gut could not be directly assessed. Future studies applying shotgun metagenomics or strain-specific qPCR assays will be required to determine the colonization dynamics of BLa80.

Despite successful randomization of clinical and demographic characteristics, statistically significant differences in baseline microbiota composition were observed between groups. These baseline differences were not adjusted for in subsequent analyses. Therefore, post-intervention comparisons should be interpreted with caution, as observed differences may reflect both intervention-associated changes and pre-existing microbial variation.

The observed decrease in total *Bifidobacterium* abundance in the probiotic group at Day 180 should be interpreted within the context of normal microbial maturation (28, 29). Multiple longitudinal cohorts have shown that *Bifidobacterium* naturally declines during the transition from infancy to toddlerhood, accompanied by expansion of taxa such as *Akkermansia, Fusicatenibacter*, and members of *Ruminococcaceae* (30). These changes reflect ecological maturation rather than dysbiosis. The probiotic group exhibited hallmark features of this maturation trajectory, including enrichment of mucin-degrading and SCFA-producing genera, which is consistent with the observed improvements in epithelial barrier markers and inflammatory tone (24, 31). Additional exploratory analyses examining associations between bifidobacterial relative abundance and clinical or immune outcomes demonstrated little definitive mediation effects. This finding may reflect the limitations of genus-level resolution inherent to 16S rRNA sequencing, which precludes strain-specific detection of BLa80. Future studies employing shotgun metagenomics or strain-specific quantification may clarify the role of probiotic engraftment and identify potential responder phenotypes. Thus, the clinical benefits of BLa80 appear to arise not from increasing total *Bifidobacterium* abundance but from steering the gut ecosystem toward a more functionally competent and immunoregulatory state (32–34). The observed reduction in richness indices (e.g., Chao1 and Fisher) likely reflects a transition toward a more specialized and functionally efficient microbial community, rather than a loss of ecological stability.

Feeding practices represent another potential influence on bifidobacterial dynamics (35, 36). Although all participants were formula-fed at enrollment, detailed longitudinal records of feeding transitions (e.g., timing and type of complementary feeding) were not collected during the 180-day follow-up. As a result, feeding mode could not be incorporated as a time-varying covariate in the analyses. However, formula feeding remains the predominant dietary pattern in this population, and prior evidence indicates that the impact of feeding mode on *Bifidobacterium* abundance diminishes after the first months of life as the gut microbiota undergoes diversification (28, 37, 38). Moreover, the functional and immunological changes observed, such as enhanced tryptophan metabolism, vitamin biosynthesis pathways, and increased sIgA, are unlikely to be explained solely by feeding patterns (39, 40). Nonetheless, this represents a potential source of unmeasured confounding and should be addressed in future studies.

The predicted functional profiling findings provide deeper insight into the metabolic consequences of these taxonomic shifts. The upregulation of tryptophan metabolism in the probiotic group is particularly relevant, as tryptophan derivatives serve as ligands for the aryl hydrocarbon receptor, a key regulator of immune homeostasis and barrier integrity (22, 41). This was complemented by enhanced fatty acid biosynthesis pathways, potentially supporting epithelial membrane integrity (42). The significant enrichment in vitamin metabolism pathways, specifically for riboflavin (B2), biotin (B7), and retinol (A), suggests that the BLa80-modulated microbiome enhances the provision of essential micronutrients that support epithelial health and immune function (43).

The convergence of taxonomic, functional, and metabolic changes is further reflected in our immunological measures. The maintenance of hBD-2 levels, coupled with increased sIgA production and decreased calprotectin, is consistent with enhanced barrier defense, adaptive mucosal immunity, and reduced inflammation in the probiotic group, consistent with previous studies showing that probiotic-driven metabolic modulation contributes to improved host immune function (44, 45). These improvements align with the observed reduction in pathogenic *Escherichia coli* infection pathways in the functional analysis, suggesting enhanced resistance to enteric pathogens. The parallel upregulation of xenobiotic degradation pathways indicates an enhanced capacity to metabolize environmental toxins, which may represent an additional protective mechanism against inflammatory triggers (46). In contrast, the placebo group's functional profile showed enrichment of less favorable pathways, including apoptosis and flagellar assembly, which may reflect ongoing microbial conflict and epithelial turnover, and biosynthesis pathways for antibiotics and secondary metabolites that could potentially influence host physiology in undesirable ways (47, 48). Statistically significant differences in microbiota composition were observed between groups at baseline, despite overall comparability in clinical and demographic characteristics. As follow-up analyses were not adjusted for baseline microbiota levels, the observed divergence at the end of the intervention should be interpreted cautiously, as it may reflect both intervention-associated changes and pre-existing microbial differences. Formal mediation analyses were not conducted; therefore, it cannot be determined whether specific microbiome or immune markers (e.g., sIgA) mediate the observed clinical effects.

This study has several notable strengths. First, it employed a rigorous randomized, double-blind, placebo-controlled design with a relatively large sample size and 180-day follow-up, providing robust evidence for the clinical effects of *Bifidobacterium animalis* subsp. *lactis* BLa80 in early childhood. Second, participants were recruited from major maternal and child health centers in Sichuan Province, representing a typical urban–suburban pediatric population in southwestern China, which strengthens the generalizability of the findings. Third, the integration of clinical outcomes with microbiome and immune data, including gut microbiota profiling, functional pathway prediction, and fecal immune markers, allowed comprehensive mechanistic interpretation linking the intervention to microbial and immunological remodeling.

The study also has some limitations. Recruitment was restricted to Sichuan Province in southwest China, which may limit the generalizability of these findings to populations with different genetic backgrounds, environmental exposures, healthcare systems, and dietary patterns. In addition, all participants were formula-fed at enrollment, and therefore the findings may not be directly applicable to breastfed or mixed-fed populations, where gut microbiota development and immune maturation may differ substantially. Future studies in more diverse geographic settings and across different infant feeding practices are warranted to confirm the broader applicability of these findings. Although baseline characteristics were balanced, detailed daily records of feeding transitions (e.g., exact timing and type of complementary foods introduced) were not collected, precluding more granular adjustment for feeding pattern as a time-varying covariate. Longitudinal microbiome analyses were not adjusted for potential confounders such as delivery mode, household registration, age, sex, and monthly household income. Although these variables were generally balanced at baseline, residual confounding cannot be excluded. Antibiotic use during the intervention period was not systematically recorded and therefore could not be accounted for in sensitivity analyses. Given the known impact of antibiotics on gut microbiota composition, this represents a potential source of unmeasured confounding. Future studies should incorporate detailed antibiotic exposure data to enable more rigorous adjustment. The use of 16S rRNA gene sequencing with V3–V4 primers did not allow strain-level resolution or specific detection of the administered *Bifidobacterium animalis* subsp. *lactis* BLa80 among resident *Bifidobacterium* populations, so direct evidence of strain engraftment could not be obtained. Finally, although the trial was adequately powered for the primary clinical endpoints, some exploratory microbiota subgroups were relatively small.

## 5 Conclusions

In conclusion, this randomized controlled trial demonstrates that early-life supplementation with *Bifidobacterium animalis* subsp. *lactis* BLa80 is a safe and effective nutritional strategy for promoting child health. The intervention significantly reduced the incidence of eczema and respiratory infections, while also alleviating a broad spectrum of symptoms related to feeding, digestion, and general irritability. The convergent clinical, immunological, and microbiome data establish that these benefits are associated with remodeling of the gut ecosystem. Specifically, BLa80 supplementation was associated with a shift toward a microbial community structure and functional capacity that may enhance gut barrier integrity, support immunoregulatory pathways, and reduce systemic inflammation. These findings provide a strong mechanistic rationale for the use of probiotic BLa80 as a preventive measure to support immune and metabolic health in the critical early years of life.

## Data Availability

The data presented in the study are deposited in the NCBI repository (https://www.ncbi.nlm.nih.gov), accession number PRJNA1464060.
