# Supplementary material for: Efficacy of Bifidobacterium lactis BLa80 in preventing early childhood eczema and respiratory infections via gut microbiome and immune modulation
Source: Front Nutr. 2026 May 28;13:1727191. doi: 10.3389/fnut.2026.1727191 (PMC13255822; doi:10.3389/fnut.2026.1727191)
Supplement: Supplementary file 1 [file Supplementary_file_1.docx]

| 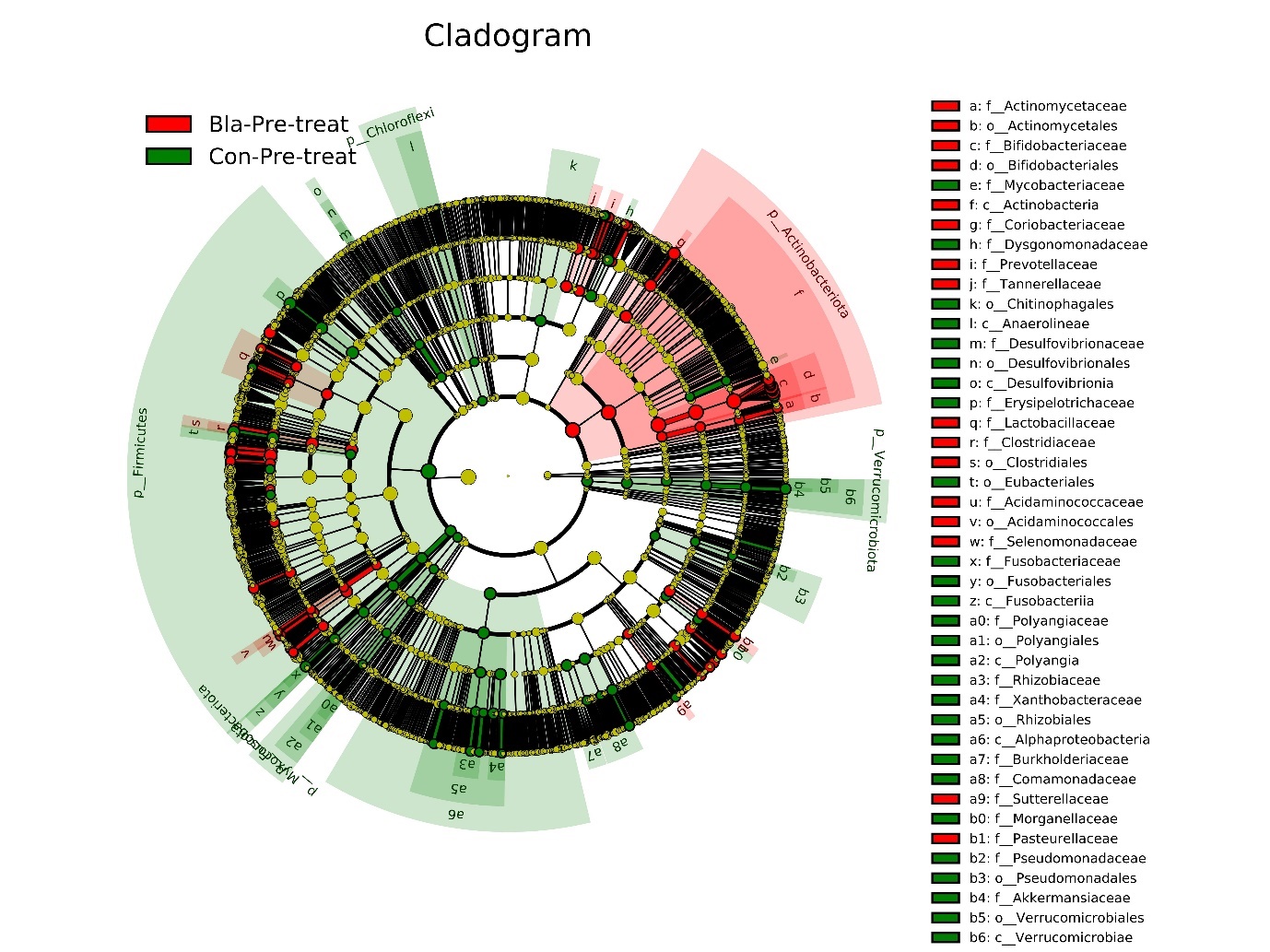 |
| --- |
| A |
| 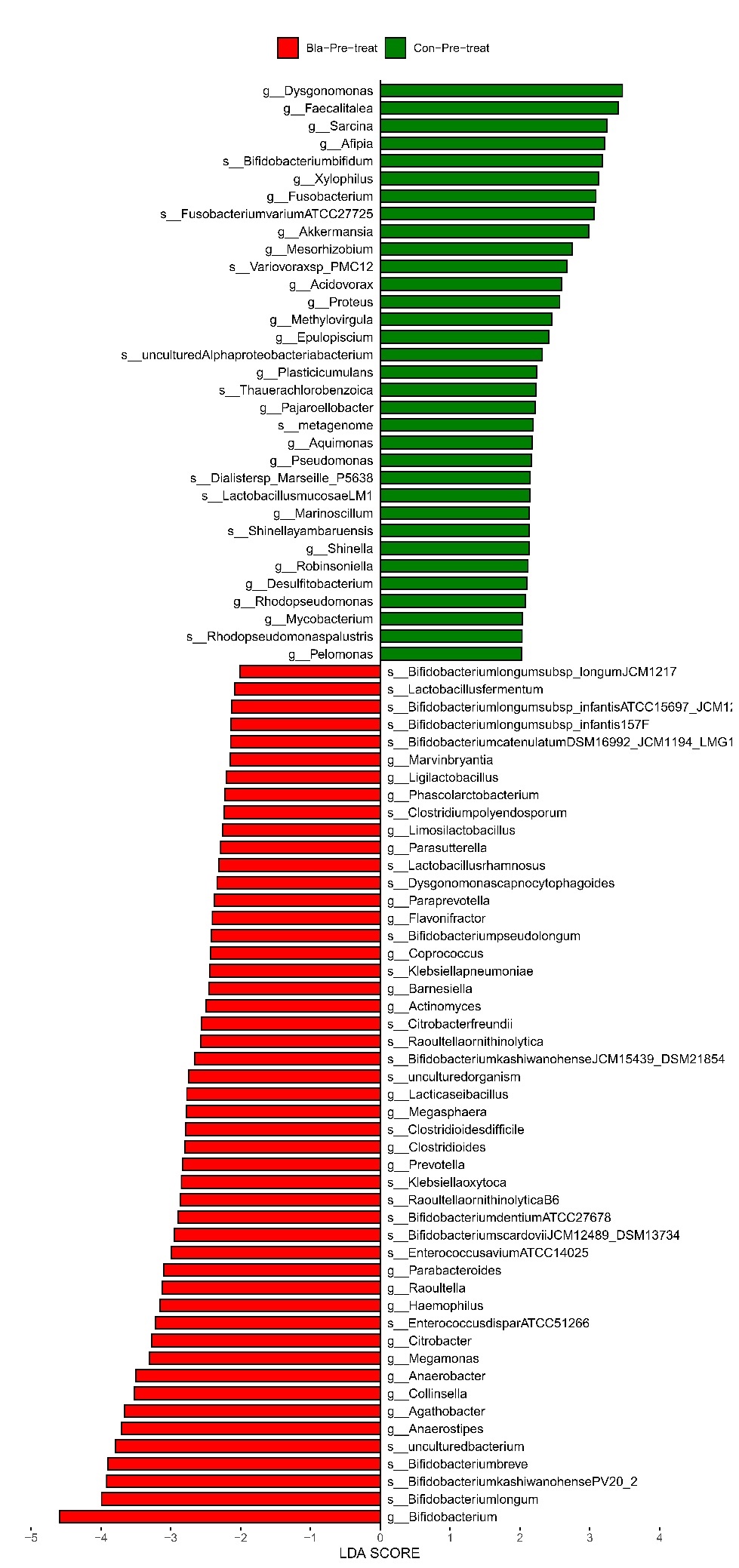 |
| B |
| 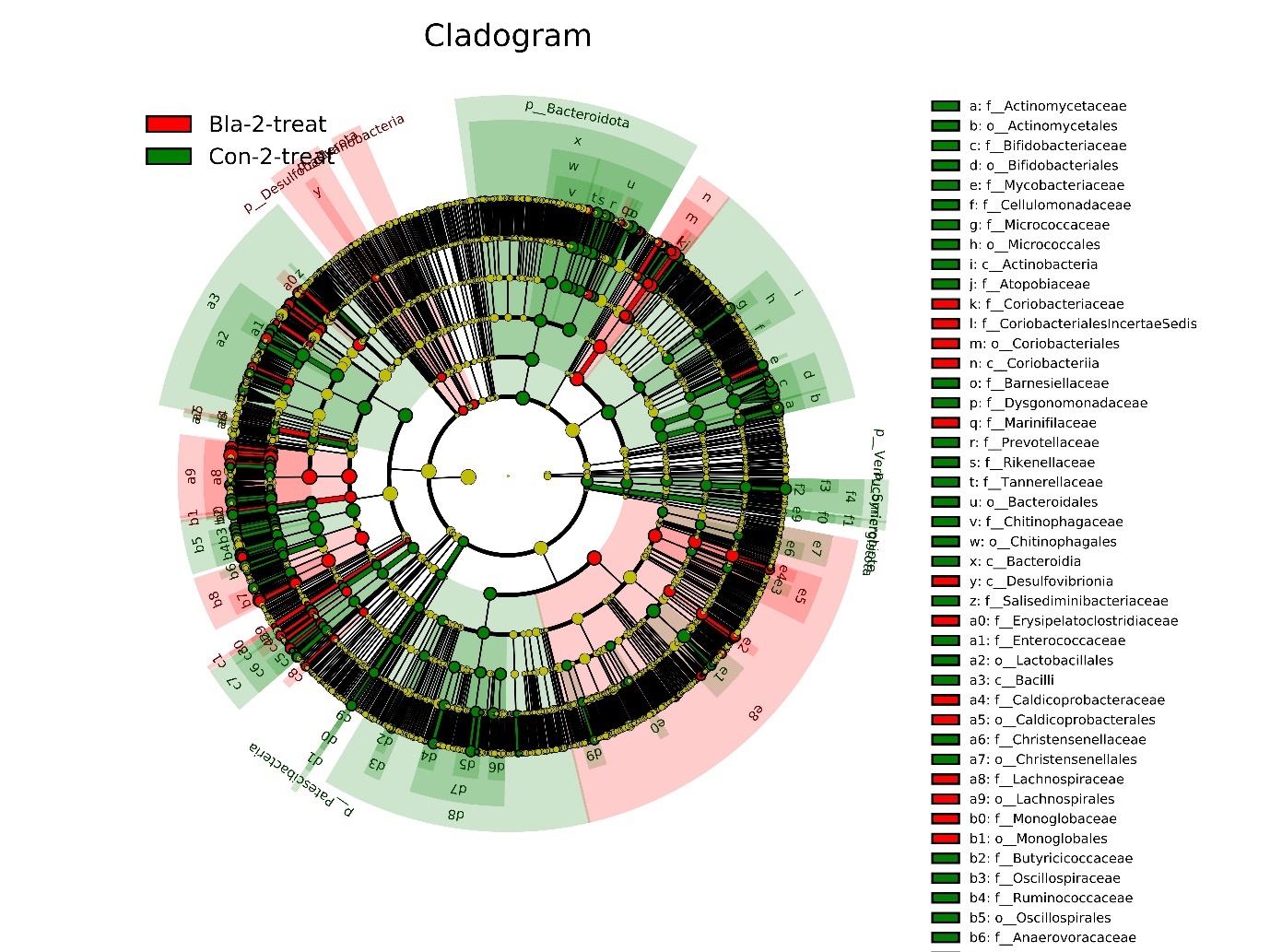 |
| C |
| 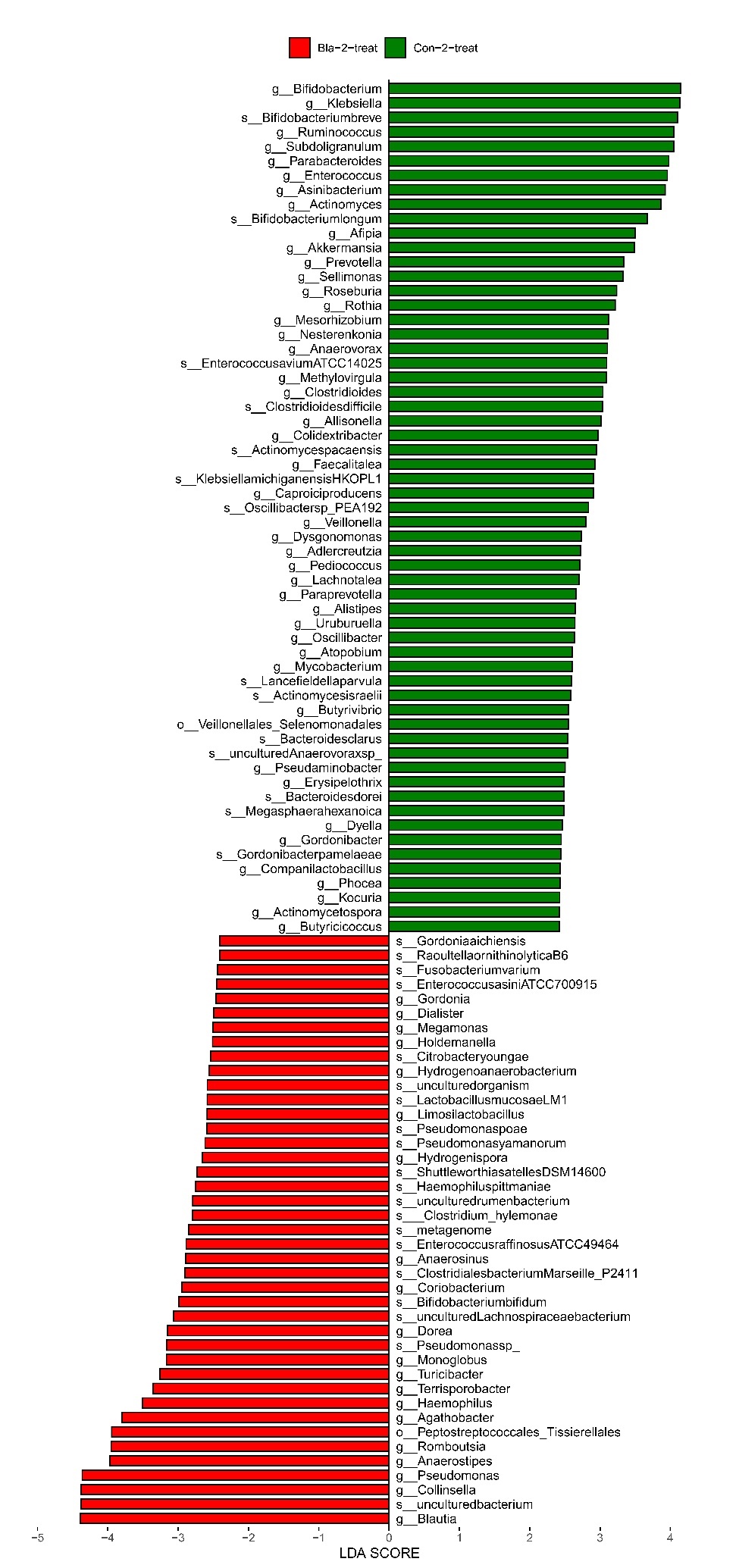 |
| D |

**Supplementary Figure 1.** LEfSe-based identification of differential gut microbiota between the probiotic gourp and the placebo group at baseline and Day 180. (A, B) Differentially enriched taxa at baseline between the probiotic group (Bla-Pre-treat, red) and the placebo group (Con-Pre-treat, green), displayed as a cladogram (A) and LDA score plot (B). (C, D) Differentially enriched taxa at Day 180 between the probiotic group (Bla-Pre-treat, red) and the placebo group (Con-Pre-treat, green). Cladograms depict phylogenetic relationships of significantly different taxa, and LDA bar plots indicate effect size and direction of enrichment. LDA threshold ≥ 2.0, *p* < 0.05. LDA = linear discriminant analysis; LEfSe = Linear Discriminant Analysis Effect Size.

| 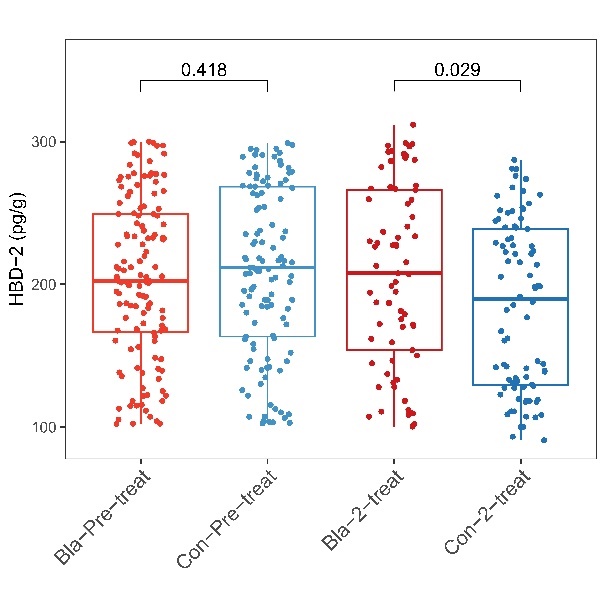 | 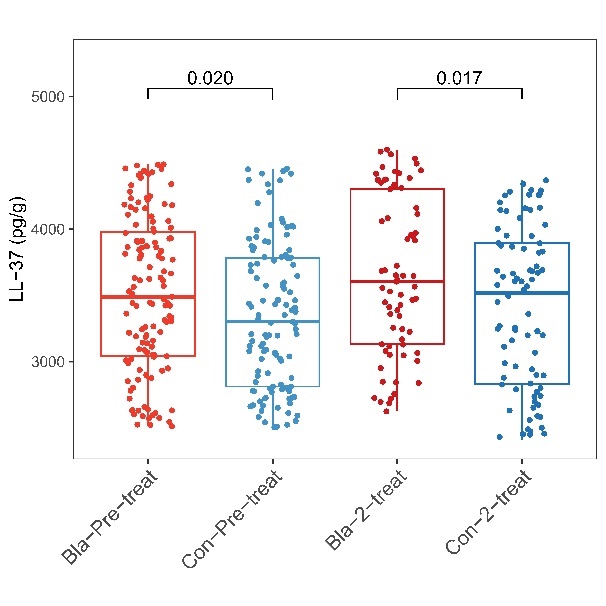 |
| --- | --- |
| A | B |
| 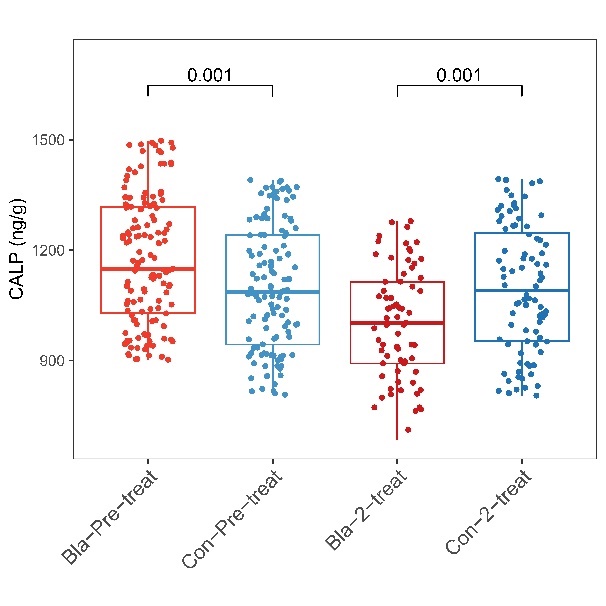 | 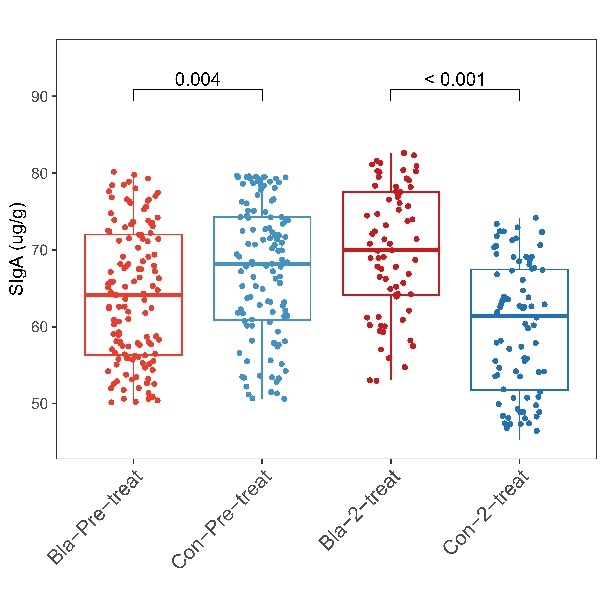 |
| C | D |

**Supplementary Figure 2.** Between-group comparisons of fecal immune markers at baseline and post-intervention. (A) Calprotectin (CALP), (B) human β-defensin 2 (hBD-2), (C) cathelicidin (LL-37), and (D) secretory immunoglobulin A (sIgA) concentrations in the probiotic and placebo groups at baseline and after the intervention period. Data are presented as boxplots showing medians and interquartile ranges; dots represent individual values. Statistical comparisons were conducted using the Mann-Whitney U test. p < 0.05 considered significant.

| 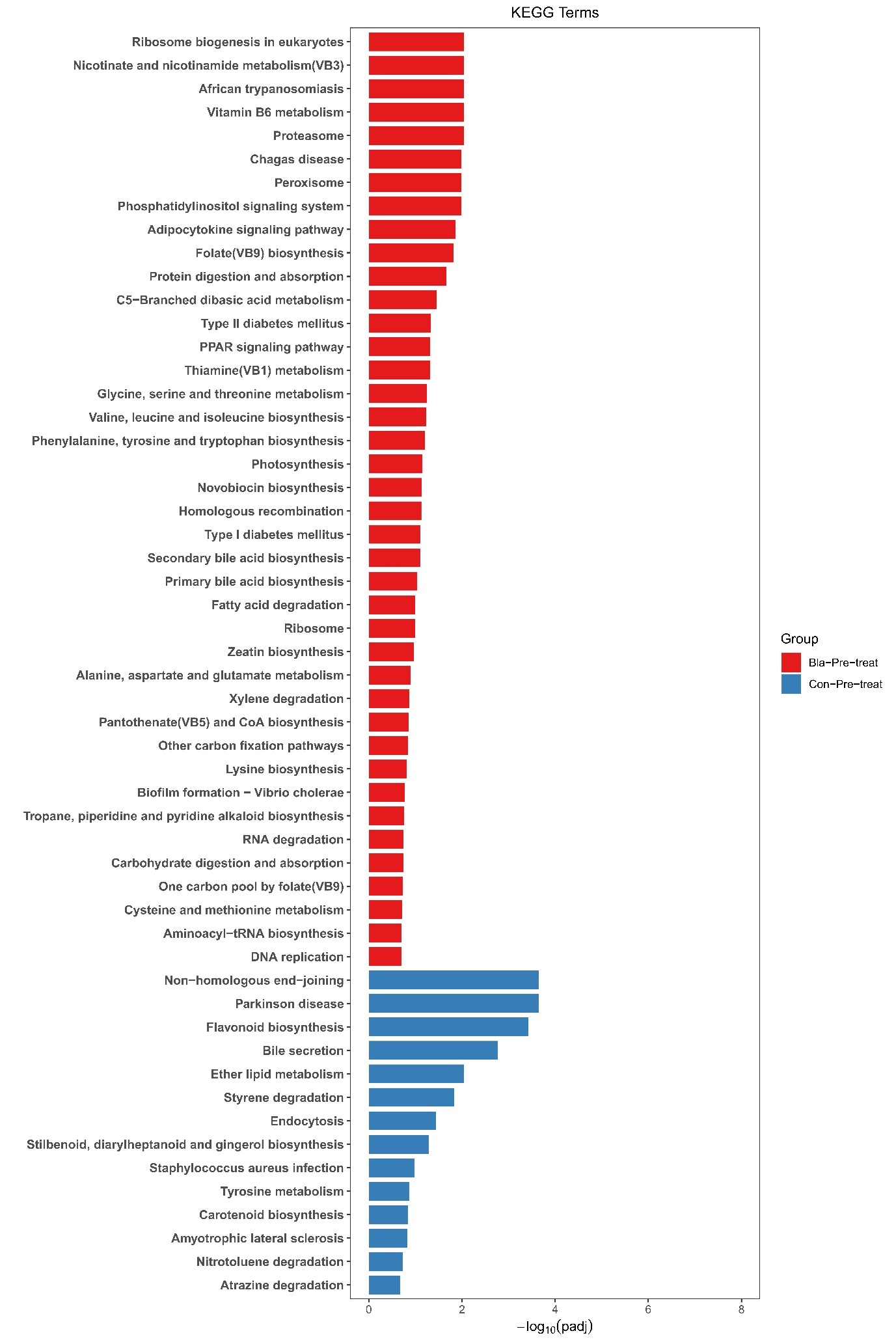 |
| --- |
| A |
| 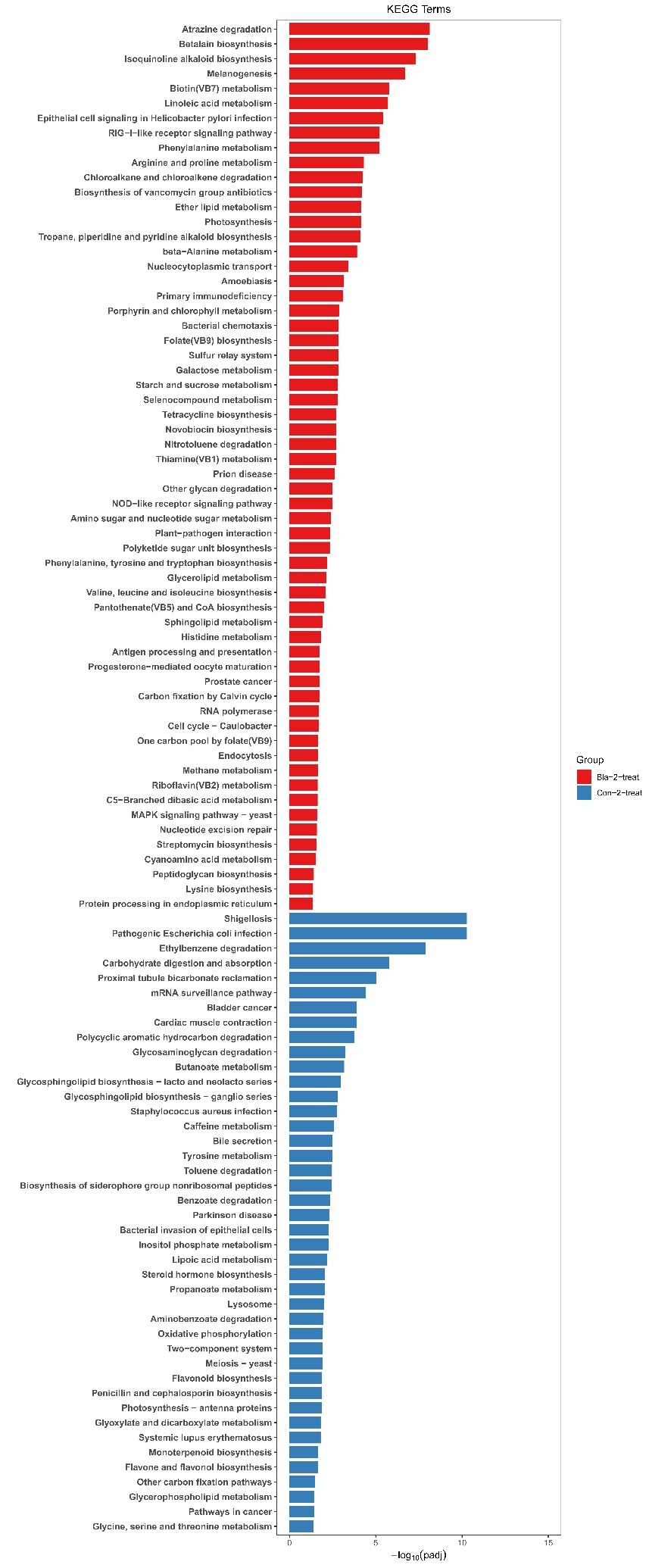 |
| B |

**Supplementary Figure 3.** KEGG functional pathway enrichment following 180 days of intervention. (A) Enriched KEGG pathways at baseline (Probiotic vs. Placebo). (B) Enriched KEGG pathways at Day 180 (Probiotic vs. Placebo). Red bars represent pathways of the probiotic group (Bla80-Pre-treat, Bla80-2-treat), and blue bars represent pathways enriched at baseline (Con-Pre-treat, Con-2-treat). Functional predictions were performed using PICRUSt2 based on 16S rRNA gene data, and KEGG pathway enrichment was visualized using STAMP. KEGG = Kyoto Encyclopedia of Genes and Genomes.

| 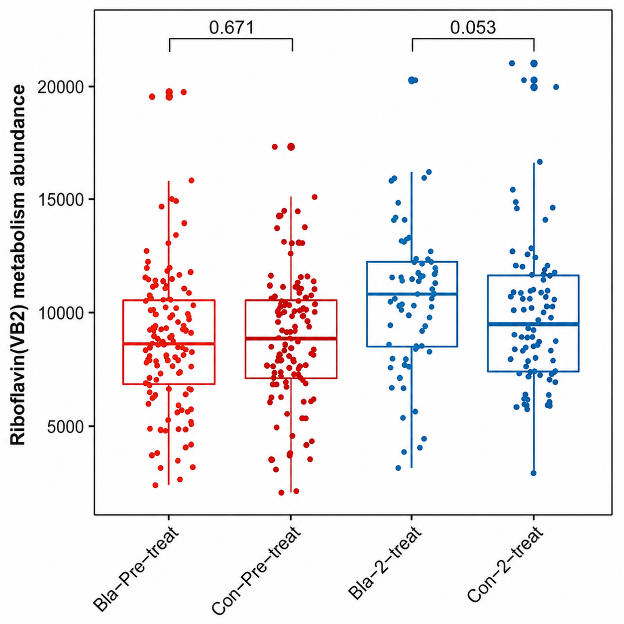 | 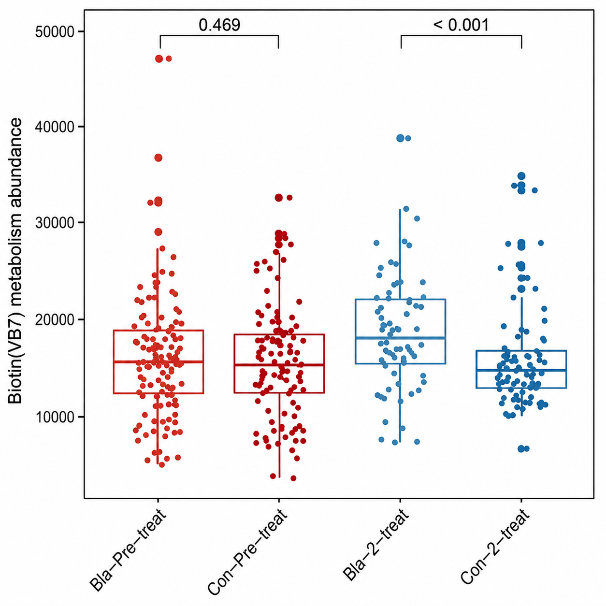 |
| --- | --- |
| A | B |
| 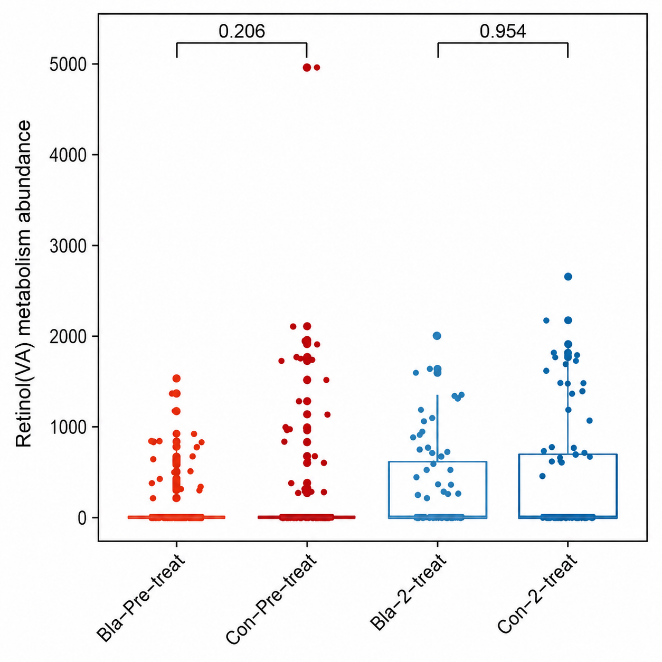 |  |
| C |  |

**Supplementary Figure 4.** Between-group comparisons of vitamin metabolism pathway regulation at baseline and post-intervention. (A) Biotin metabolism, (B) riboflavin metabolism, and (C) retinol metabolism pathway regulation in the probiotic and placebo groups at baseline and after the intervention period. Data are presented as boxplots showing medians and interquartile ranges; dots represent individual values. Statistical comparisons were conducted using the Mann–Whitney U test. p < 0.05 considered significant.
